# Mapping rootable depth and root zone plant-available water holding capacity of the soil of sub-Saharan Africa

**DOI:** 10.1016/j.geoderma.2018.02.046

**Published:** 2018-08-15

**Authors:** Johan G.B. Leenaars, Lieven Claessens, Gerard B.M. Heuvelink, Tom Hengl, Maria Ruiperez González, Lenny G.J. van Bussel, Nicolas Guilpart, Haishun Yang, Kenneth G. Cassman

**Affiliations:** a ISRIC - World Soil Information, PO Box 353, 6700 AJ, Wageningen, The Netherlands; b ICRISAT - International Crop Research Institute for the Semi-Arid Tropics, PO box 39063, Nairobi, Kenya; c IITA - International Institute of Tropical Agriculture, PO Box 10, Duluti, Arusha, Tanzania; d Soil Geography and Landscape Group, Wageningen University & Research, PO box 47, 6700 AA, Wageningen, The Netherlands; e Environmental Systems Analysis, Wageningen University & Research, PO box 47, 6700 AA, Wageningen, The Netherlands; f Plant Production Systems, Wageningen University & Research, PO box 430, 6700 AK, Wageningen, The Netherlands; g UMR 211 Agronomie, Department SIAFEE, AgroParisTech, Thiverval-Grignon, France; h Department of Agronomy and Horticulture, University of Nebraska-Lincoln, PO Box 830915, Lincoln, NE 68583-0915, United States

**Keywords:** AfSP, Africa Soil Profiles database, AfSS, Africa Sentinel Sites database, BD, bulk density, CEC, cation exchange capacity, DSM, digital soil mapping, EC, electrical conductivity, FC, field capacity, ISFM, Integrated Soil Fertility Management, PAWHC, plant-available water holding capacity, pF, logarithm of the negative hydrostatic head or matrix potential, PWP, permanent wilting point, PTF, pedotransfer function, RI, rootability index, RZD, root zone depth (rootable depth), RZ-PAWHC, root zone plant-available water holding capacity, SFEF, soil fine earth fraction, SSA, sub-Saharan Africa, VMC, volumetric moisture content, Root zone depth, Rootability, Soil water, Digital soil map, Soil data, Sub-Saharan Africa, Maize

## Abstract

In rainfed crop production, root zone plant-available water holding capacity (RZ-PAWHC) of the soil has a large influence on crop growth and the yield response to management inputs such as improved seeds and fertilisers. However, data are lacking for this parameter in sub-Saharan Africa (SSA). This study produced the first spatially explicit, coherent and complete maps of the rootable depth and RZ-PAWHC of soil in SSA. We compiled geo-referenced data from 28,000 soil profiles from SSA, which were used as input for digital soil mapping (DSM) techniques to produce soil property maps of SSA. Based on these soil properties, we developed and parameterised (pedotransfer) functions, rules and criteria to evaluate soil water retention at field capacity and wilting point, the soil fine earth fraction from coarse fragments content and, for maize, the soil rootability (relative to threshold values) and rootable depth. Maps of these secondary soil properties were derived using the primary soil property maps as input for the evaluation rules and the results were aggregated over the rootable depth to obtain a map of RZ-PAWHC, with a spatial resolution of 1 km^2^. The mean RZ-PAWHC for SSA is 74 mm and the associated average root zone depth is 96 cm. Pearson correlation between the two is 0.95. RZ-PAWHC proves most limited by the rootable depth but is also highly sensitive to the definition of field capacity. The total soil volume of SSA potentially rootable by maize is reduced by one third (over 10,500 km^3^) due to soil conditions restricting root zone depth. Of these, 4800 km^3^ are due to limited depth of aeration, which is the factor most severely limiting in terms of extent (km^2^), and 2500 km^3^ due to sodicity which is most severely limiting in terms of degree (depth in cm). Depth of soil to bedrock reduces the rootable soil volume by 2500 km^3^, aluminium toxicity by 600 km^3^, porosity by 120 km^3^ and alkalinity by 20 km^3^. The accuracy of the map of rootable depth and thus of RZ-PAWHC could not be validated quantitatively due to absent data on rootability and rootable depth but is limited by the accuracy of the primary soil property maps. The methodological framework is robust and has been operationalised such that the maps can easily be updated as additional data become available.

## Highlights

• Rootable depth & root zone plant-available water holding capacity mapped for SSA
• Average rootable depth is 96 cm and associated RZ-PAWHC is 74 mm.
• Rootable volume reduced by a third due to restrictive aeration, sodicity, bedrock
• Accuracy rootable depth map cannot be validated due to lack of data on rootability.
• Robust framework; maps can be updated as extra data become available.

## 1 Introduction

Substantial and sustainable increases in crop yields are needed in sub-Saharan Africa (SSA) to help meet food demand due to population and income growth (Jayne et al., 2010; Pretty et al., 2011; Garnett and Godfray, 2012; van Ittersum et al., 2016). Yield increases require improved crop and soil management practices, including improved seeds and cost-effective application of nutrients in the form of organic and/or inorganic fertilisers according to the principles of Integrated Soil Fertility Management (ISFM) (Vanlauwe et al., 2010). However, ISFM will only be adopted by smallholder farmers, which make up 65–80% of the population in SSA, if the return on investment is appreciable and without too much risk. Indeed, farmer's motivation and decision making relies heavily on the perceived likeliness of obtaining a profitable return at minimized risk. This likeliness largely depends on the yield response to inputs, both in terms of magnitude and stability (i.e. temporal variation), which depends to a large extent on site-specific soil properties and year-to-year variation in weather. Hence quantitative estimates of the yield response to inputs at a given location, and especially its temporal variation, are essential for estimating the risks associated with these investments and such information may well be key to achieving higher rates of adoption of ISFM practices and especially fertiliser application (Marenya and Barrett, 2007; Dercon and Christiaensen, 2007; Rötter and van Keulen, 1997; Hiebert, 1974).

Rainfed crop production is practiced on >95% of existing farmland in SSA (Alexandratos and Bruinsma, 2012) where current average farm yields for the major cereal crops are only about 20% of the potential rainfed yields without limitations from nutrients or pests and diseases (van Ittersum et al., 2016). This potential yield represents the crop demand for nutrients and sets a reference for determining the degree that soil supply of nutrients is deficient. The amount of water available to support crop growth in these rainfed systems is largely determined by rainfall amount and timing, and the amount of water that can be stored in the soil profile and that is available for uptake by crop roots -hereafter called the root zone plant-available water holding capacity (RZ-PAWHC). The RZ-PAWHC represents a reservoir from which crops can take up water and which buffers against water deficits in periods when rainfall does not meet crop water demand and also determines the length of the growing period at the end of the rainy season in monsoonal tropical climates, and thus the appropriate cultivar to use (e.g. FAO, 1978; Zingore et al., 2007). Therefore a larger RZ-PAWHC reduces risk of drought stress and contributes to higher yields and yield stability, and thus increases the resource use efficiency (de Wit, 1992) and the probability of obtaining a profitable response to ISFM.

Data on RZ-PAWHC are thus key input to soil moisture models such as GLEAMS (Martens et al., 2017), crop growth models such as WOFOST (van Diepen et al., 1989), LINTUL (Spitters and Schapendonk, 1990), DSSAT (Jones et al., 2003), Hybrid-Maize (Yang et al., 2004) and data mining (Jeong et al., 2016; You et al., 2017) and therewith to yield gap analysis for performing *ex ante* assessments of yield responses to inputs across a wide range of environmental conditions (Grassini et al., 2015; van Ittersum et al., 2013). While recent initiatives, e.g. the Africa Soil Information Service (AfSIS) project (http://africasoils.net), have improved the availability, accuracy and resolution of spatially explicit and coherent data on soil fertility parameters in SSA (ISRIC, 2013; Hengl et al., 2015b, Hengl et al., 2017b), there are few data on RZ-PAWHC or root zone depth. This study, which is a collaborative initiative of the Global Yield Gap and water productivity Atlas (GYGA) project (www.yieldgap.org) and the AfSIS project, attempts to fill this “data gap” by developing the first spatially explicit soil maps for SSA of root zone depth and RZ-PAWHC. In this study we derive maps for maize as a reference crop because maize is an important cereal in SSA and to a large extent representative for other major cereals.

## 2 Materials and methods

### 2.1 Definitions and methodological framework

The RZ-PAWHC reflects the adequacy (capacity) of soil to store water and support crop growth when rainfall is insufficient to meet crop water requirements. RZ-PAWHC (expressed by an absolute value (mm)) is composed of three components which are aggregated to a single parameter. The first component is the plant-available water holding capacity (PAWHC) of the soil fine earth and is defined as the amount of soil moisture retained over the range in which the soil is neither too wet nor too dry for crop roots to take up soil water. The PAWHC is assessed per depth interval and expressed as a volumetric fraction. The second component is the soil fine earth fraction (SFEF) which is the volume of soil fine earth (particle size < 2 mm) as a fraction of the volume of soil whole earth. The SFEF determines the net volume of soil, per depth interval, that can retain soil moisture and that crop roots can effectively exploit. The third component is the total depth interval from which the crop can extract water, which is the rootable soil depth or root zone depth (RZD). This study derives maps of the RZ-PAWHC for maize which has a genetically defined potential root zone depth, attained near anthesis, between 100 and 170 cm (van Keulen and Wolf, 1986). In this study, a maximum potential root zone depth of 150 cm is used.

**Fig. 1:**
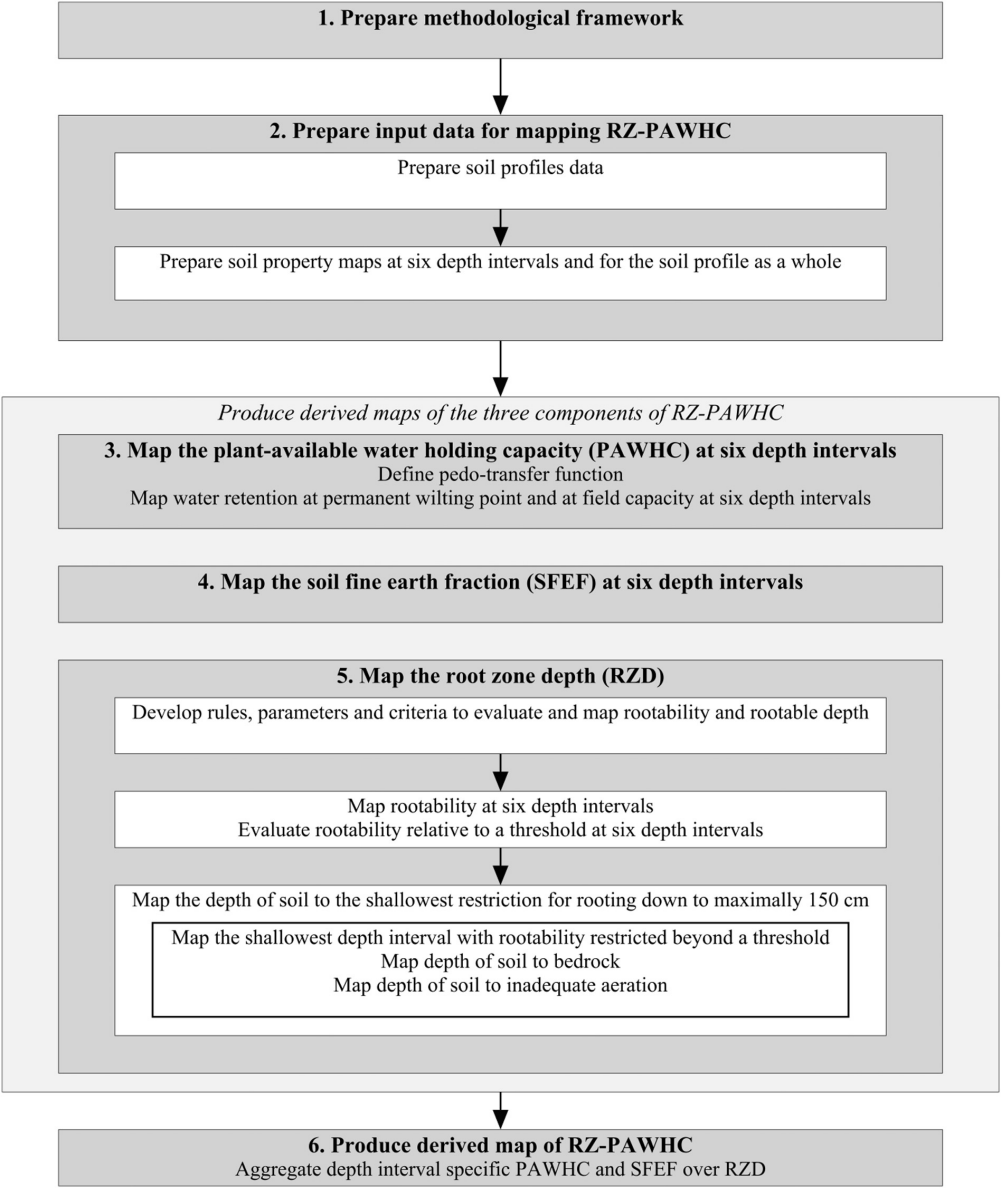
Overview of the methodological framework to map RZ-PAWHC.

There are three main ways to map each of the three components defining RZ-PAWHC. The first is to collect sufficient direct observations of the three soil properties, and use these primary soil profile data for producing interpolated maps, either representing individual soil profile layers or the soil profile as a whole. This direct approach can make use of digital soil mapping (DSM) techniques such as regression kriging and machine-learning (McBratney et al., 2003; Hengl et al., 2004, Hengl et al., 2015b; Lagacherie et al., 2006) and requires sufficient data well distributed over geographic- and feature space. The second way is to infer secondary soil profile data for the three targeted soil properties from primary soil profile data readily available for other soil properties, e.g. by existing or yet to be established pedotransfer functions (PTF; Bouma, 1989), and to use the derived data and DSM techniques to produce interpolated maps of each of the three target soil properties (first calculate, then interpolate; Heuvelink and Pebesma, 1999). This approach requires the available soil profile data to be sufficiently coherent in terms of scope, homogeneity and completeness, without important data gaps, to consistently derive the secondary data. The third way is to first create interpolated soil property maps, using DSM and primary soil profile data which are available in sufficient quantities and of sufficient coherence, and then use these interpolated coherent maps as input for (pedotransfer) functions, rules and criteria to calculate derived, inferred, maps of the targeted secondary soil properties (first interpolate, then calculate). For each of the three ways, the results for different depth intervals for water retention and the soil fine earth fraction can be aggregated into a single value over the rootable soil depth to produce the RZ-PAWHC map. Because the soil profile data available for this study were not complete for all required variables, and the soil depths sampled were not consistent and often did not include soil layers below 50 cm depth, this third approach was used in this study. Basically, this approach is a digital soil assessment (Minasny et al., 2012). An overview of the methodological framework to map RZ-PAWHC is given in Fig. 1. The steps in the workflow are explained in detail in the next sections.

### 2.2 Data preparation

#### 2.2.1 Soil profiles data

Soil profiles data used for mapping and validation, and for the development and testing of pedotransfer functions and rules to produce derived data and maps, came from two soil profile datasets generated by the AfSIS project. First, the Africa Soil Profiles database (AfSP) which is a compilation of georeferenced and standardised legacy soil profile data for SSA (Leenaars et al., 2014a) and is available at www.isric.org/projects/africa-soil-profiles-database-afsp. The AfSP version 1.2 consists of soil data taken at 18500 profile point locations which are described and sampled on average at 4.1 (±1.6) depth intervals to an average soil depth of 125 (±65) cm. The second soil dataset was collected more recently from 60 sentinel sites of 10 × 10 km (AfSS) and is available at afsisdb.qed.ai with data for 9600 point locations sampled at the 0–20 and 20–50 cm depth intervals. Ten percent of the AfSS data is the result of direct measurements and the other 90% is inferred from spectroscopic data (Sila et al., 2014). Adding to these two datasets were data on depth of soil to an iron pan (3660 virtual profiles) as interpreted from legacy soil maps (Boulet and Leprun, 1969) and georeferenced from polygon centroids.

Compiling soil datasets from different sources enhances the data availability but also causes some degree of heterogeneity (Leenaars et al., 2014b; Hendriks et al., 2016). This can cause incompatibilities in producing soil maps or estimating pedotransfer functions but also adds value in other ways. The datasets show overlap in terms of recorded soil properties, such as particle size fractions, pH, electric conductivity, exchangeable cations and the contents of organic carbon, nitrogen and available phosphorus, but the field and laboratory procedures used to assess these properties differ. These differences required careful querying of the recorded procedures to compile the data under a common standard. Besides overlap, the datasets also show important differences in terms of recorded soil properties, for details see Leenaars et al. (2015) or Hengl et al. (2015b). Measured and inferred data on the contents of extractable elements, including micro-nutrients, were predominantly available from the AfSS dataset while measured data on bulk density, cation exchange capacity, water retention, coarse fragments content and, though few, root presence as well as data on depth and drainage of the soil profile were available only from the AfSP database. Added value was created by combining the recent AfSS data, explaining short distance variability of some soil properties at shallow depth, with the generally older AfSP data, explaining large distance variability of, both similar and other, soil properties at larger depth.

#### 2.2.2 Soil property maps

AfSoilGrids250m (Hengl et al., 2015b) was used for this study. AfSoilGrids250m is a coherent collection of gridded soil property maps of SSA which were produced in the context of the AfSIS project, available at www.isric.org/projects/soil-property-maps-africa-250-m-resolution. The maps were created in 3D from soil profiles data and maps of explanatory variables (“covariates”), including depth covariates, by using machine learning (random forests) as the DSM technique to model the trends and ordinary kriging to interpolate the residuals. For details of the function, implemented in the GSIF package for R (Global Soil Information Facilities), see Hengl et al. (2015a). The maps have a spatial resolution of 250 m and report estimated soil property values at six standard depth intervals (i.e. 0–5 cm, 5–15 cm, 15–30 cm, 30–60 cm, 60–100 cm and 100–200 cm), matching the GlobalSoilMap specifications (Arrouays et al., 2014), for sand, silt and clay fractions, bulk density of the soil fine earth, organic carbon content, cation-exchange capacity, sum of exchangeable bases, exchangeable acidity (aluminium) and pH-H_2_O. AfSoilGrids250m does not include maps of the uncertainties associated with the maps of soil property estimates and it was beyond the scope of this study to produce such maps.

Additional soil property maps were produced and cross-validated following the methodology of SoilGrids250m. Maps were created of the coarse fragments content, exchangeable sodium content and electric conductivity, at six depth intervals, and of the soil profile drainage class and depth to bedrock. These additional properties were difficult to predict accurately because of inadequately queried input data of sometimes subjective imprecise nature (coarse fragments class, drainage class) and high degree of skewness and the maps showed few obvious errors at first sight. This was also true for the map of bulk density, for which relatively few data were available. A second iteration was made to create maps of the additional soil properties as well as of bulk density using newly queried soil profiles data and additional covariates. Newly queried from the soil profile data were data on bulk density measured from the oven-dry soil fine earth excluding data measured from the soil whole earth thus excluding coarse fragments (e.g. DRC, 1967) and data on electric conductivity measured in the unsaturated extract (EC) excluding data measured in the saturation extract (ECe). Added to the data for the top 50 cm on sodium, inferred from spectroscopic data with the inference being particularly poor for sodium, were data on exchangeable sodium from the AfSP database measured over full profile depths. Added covariates, expected to enhance model performance, were the soil atlas of Africa (Jones et al., 2013) and maps of surficial lithology and land surface forms (USGS Rocky Mountain Geographic Science Center, 2009), groundwater table depth (Fan et al., 2013) and the annual water balance which was calculated from annual precipitation (Africa Soil Information Service (AfSIS), 2013) and annual potential evapotranspiration (Trabucco and Zomer, 2009). Maps of soil pH-H_2_O, sum of exchangeable bases and clay content were added as covariates to support the predictions of exchangeable sodium, for details see Leenaars et al. (2015). The resulting maps were validated according to the procedures described by Hengl et al. (2015b) using 5–fold cross-validation where each model was re-fitted five times using 80% of the profiles data and then applied to predict at the remaining 20% of profiles. Predictions were then compared with the put-aside observations (including observations inferred from spectroscopic data which will affect the cross-validation). Calculated were the Root Mean Squared Error (*RMSE*) and the amount of variation explained by the model, derived as ∑_%_ = 100 ∗ [1 − (*SSE/SST*)], where *SSE* is the sum of squared errors at the cross-validation points (i.e. *RMSE*^2^ · *n*), and *SST* is the total sum of squares of the original observations.

From these primary soil property maps, resampled from 250 m to 1 km, maps of the three components defining RZ-PAWHC were derived. This will be explained in the next sections.

### 2.3 Mapping plant-available water holding capacity of the soil fine earth (PAWHC)

The PAWHC is defined, for a given soil depth interval, as the difference between the volumetric moisture content (VMC) of the soil fine earth at field capacity (VMC-FC) and at permanent wilting point (VMC-PWP). Note that this definition excludes the volume of soil occupied by gravel, stones and other coarse fragments. While the PWP is crop-specific, it is commonly defined and valid for maize as the moisture potential of the soil equal to pF 4.2, which is equivalent to a suction of 15,000 cm. FC is the situation when wet soil is freely drained but the corresponding soil moisture potential is not strictly defined and commonly varies between pF 1.7 to pF 2.5, i.e. a moisture potential of 50 to 300 cm, due to differences in soil matrix configuration. Gijsman et al. (2007) define FC for coarse, medium and fine textured soils at respectively pF 2.0, 2.3, 2.5 (i.e., 100, 200, 300 cm). For the purpose of producing maps of PAWHC, it was decided not to define FC differently for different textures because the results in a 3D configuration (with textures varying across different positions and depth intervals), would become highly inconsistent as concluded from tests applied to the soil profiles data. Instead, each of the three definitions for FC has been applied, irrespective of texture, to calculate the corresponding PAWHC and the significance of the definition of FC on PAWHC was evaluated.

Data on soil water retention, measured at various water potentials including FC and PWP, and also saturation, were available from the AfSP database for approximately 2500 soil profiles (8000 layers). This amount was considered insufficient to support the production of directly interpolated maps of VMC and of PAWHC for SSA. Data on soil water retention as recorded in the AfSS dataset had been calculated from primary data using a pedotransfer function (PTF) based on Brooks and Corey (1966). Instead we used a PTF specifically developed for tropical soils (Hodnett and Tomasella, 2002) which parameterises the van Genuchten (1980) equations and which was validated by Wösten et al. (2013) on the basis of the measured soil profile data from the first version of the AfSP database (Leenaars, 2012). This PTF requires data on sand, silt and clay contents, organic carbon content, bulk density, cation exchange capacity and pH-H_2_O, with the latter two included as proxies to account for the mineralogy (kaolinite) of highly leached tropical soils.

Maps were available for each of these soil properties and were used as input to the PTF to compute water retention maps for each of the six standard depth intervals, including maps of the VMC at PWP (pF 4.2) and at FC (pF 2.0, 2.3 and 2.5) and corresponding maps of the PAWHC. Using the newly produced maps for bulk density as new input, water retention maps were computed again, of VMC at PWP and at FC (defined at pF 2.3), and PAWHC was calculated applying this single specification of FC. Also computed were maps of VMC at saturation (pF 0.0). The resulting maps were validated by comparing the mapped values with the observed values, per depth interval, and reported are the amount of variation explained (*R*^*2*^) and the Mean Error (*ME*), Mean Absolute Error (*MAE*), Root Mean Squared Error (*RMSE*) and Root Median Squared Error (*RmdSE*). We computed Pearson correlation coefficients to assess the sensitivity of PAWHC for each of the soil properties mapped and included in the PTF.

### 2.4 Mapping the soil fine earth volume as fraction of the soil whole earth volume (SFEF)

The volume of soil fine earth (particle size < 2 mm) is a fraction of the volume of the soil whole earth excluding the volume of coarse fragments. Maps of the soil fine earth volume were derived for each of the six standard depth intervals from the maps of the volumetric coarse fragments content (v%) deducted from 100%. The maps of coarse fragments content were produced by DSM using data from >40,000 soil layers of approximately 10,000 soil profiles. Note that the majority of these data were derived from descriptive class values as collected from field observations and consequently these data are not very precise and neither can be the interpolated maps (which are validated as previously described).

### 2.5 Mapping the rootable soil depth (RZD)

#### 2.5.1 Definitions and evaluation framework

The depth interval defining the soil volume accessible to plants (and determining the RZ-PAWHC) is determined by root zone depth (RZD), also commonly referred to as the rootable soil depth, the effective (plant exploitable) soil depth (GlobalSoilMap, 2015; Arrouays et al., 2014) or the root restricting (i.e. plant accessible) soil depth (Soil Survey Division Staff, 1993). The latter is defined as the depth at which root penetration is strongly inhibited with the restriction defined as the inability to support more than very few fine or few very fine roots. We defined RZD from a gradual scalable phenomenon to an abrupt and unscaled one by assuming rootability as fully unrestricted (adequate, suitable) within rootable depth and fully restricted (inadequate, unsuitable) beyond rootable depth. This assumption is justified as diffusivity, in soil nearly as dry as wilting point, is generally so high that small gradients in water content suffice to transport water to the -few fine- roots at required rate (de Willigen and van Noordwijk, 1987). Rootability and rootable depth are not directly reflected by any soil property which can be observed during soil field studies if not by obvious properties such as a laterite pan or by actual root density and depth itself. From the AfSP database, observed data on presence or absence of roots in about 2500 soil profiles (8500 layers) are available. Rooted depth, not rootable depth, was recorded for some 4000 profiles. These data represent momentary observations of roots of a wide variety of vegetation types (not of maize at anthesis specifically) and were considered too heterogeneous to produce interpolated maps from using DSM. Instead, rootability was derived and mapped from a number of relevant soil factors which could be robustly parameterised and inferred from mapped or map-able soil properties and which are soil-intrinsic and thus not easily altered through management or dynamically varying conditions (thus excluding factors such as penetration resistance). Two types of soil factors were defined based on these considerations, including factors to evaluate the individual soil layers separately (adequacy of porosity, volume, textural configuration, cementation, acidity, alkalinity, sodicity, salinity, toxicity and morphology) and factors to evaluate the soil profile as a whole (depth of aerated soil and depth to bedrock). Rules to infer rootability were established and parameterised purely from literature sources due to the absence of data needed to newly develop and calibrate such rules. This evaluation framework is basically a land evaluation procedure (FAO, 1976) in which soil factors, corresponding with so called land qualities inferred from land characteristics, are compared with land use requirements and expressed as adequacies (suitability) of the soil relative to the requirements of the crop.

#### 2.5.2 Mapping the rootability of soil layers relative to a threshold

Rootability of each soil depth interval (soil layer) was evaluated by soil factors parameterised by a rootability index (RI). The RI expresses the adequacy (0–100%) of each soil factor to support root growth relative to optimal root growth. This scalable approach was adapted from Driessen and Konijn (1992), based on Kiniry et al. (1983) and Rijsberman and Wolman (1985). Its scalability (0–100%) was made unscaled (0 or 100%) by defining a threshold index for each soil factor, which assumes rootability as fully restricted (inadequate) at RI below the threshold index and fully unrestricted (adequate) at RI above the threshold index. This threshold index was set at 20% for all identified soil factors, based on Jones (1983), and a soil layer is assumed to be inadequate for rooting if one or more of the soil factors are evaluated beyond this threshold index (<20%).

**Fig. 2:**
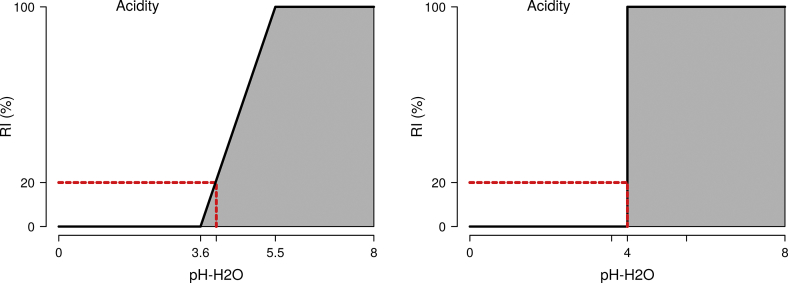
Illustration of a). a scalable rule to evaluate the adequacy of a soil factor (acidity), expressed by a rootability index (0–100%), depending on a soil layer property (pH-H_2_O), b). the rule is made unscaled by a threshold index (20%) relative to which soil is considered either fully adequate (100%) or fully restrictive (0%) to rooting.

Based on literature review, rules were developed to enable evaluation of ten selected soil factors (porosity, volume, textural adequacy, cementation, acidity, alkalinity, salinity, sodicity, toxicity and morphology). This included the identification and parameterisation of soil properties (e.g. pH-H_2_O) relative to the RI for each of the soil factors (e.g. acidity) and definition of the property values at the threshold index value of 20%. Fig. 2 illustrates the rules developed for evaluating the adequacy of soil factors, expressed by RI's (rootability indices), depending on soil property values and also illustrates the scalable approach made unscaled by a threshold index and associated threshold property value.

Details about the process used to identify and parameterise soil properties, relative to the rootability index, for each of the soil factors are described in Leenaars et al. (2015), and a brief description of the major considerations is provided below. The outcomes are given in the Results section.

1. *Porosity* determines the space available for roots to elongate. Reduced pore volume causes physical resistance to root penetration. Measured data or maps of pore volume, or of -moisture dependent- penetration resistance, were not available and instead two parameters that serve as a proxy were used, namely, the volumetric moisture content at saturation (VMC-Sat) and the bulk density as a function of clay content (f.BD). VMC-Sat was considered equal to pore volume, and it was calculated and mapped using the PTF for assessing water retention. Bulk density (BD) reflects the combined volumes and weights of both air and fine particles in the soil. At a given bulk density, pore volume is large if the soil is sandy and low if clayey because of the particle density (PD) of sand exceeding that of clay (assumed 2.65 and 2.10 kg/dm^3^, respectively). With PD specified as a function of clay content, pore volume (PV) depends on BD, as PV = 100 × (1 − (BD / PD)). This we simplified into a single variable (f.BD) which evaluates BD relative to a critical, texture-dependent, BD. The parameterisation to assess RI's from VMC-Sat and f.BD was derived from Kiniry et al. (1983), GlobalSoilMap (2015), Hazelton and Murphy (2007), Rijsberman and Wolman (1985), FAO (2006) and Jones (1983). Suboptimal conditions for rooting (RI < 100%) occur when porosity is <40 v% (Landon, 1991) and the rootability threshold (RI = 20%) is when porosity is 30 v%.
2. *Soil volume* is insufficient for roots to proliferate and establish optimally if soil is dominated by coarse fragments (Rijsberman and Wolman, 1985; Sanchez et al., 1982, Sanchez et al., 2003) which corresponds to a volumetric content of coarse fragments exceeding 80% (FAO, 2006). Maps of the coarse fragments content were produced as previously described.
3. *Textural adequacy* for rooting was derived from two soil properties, i.e. sand content and the abruptness of textural change over depth. Near pure, clean sand is inhibitive to root development (Arrouays et al., 2014; GlobalSoilMap, 2015). An abrupt textural change over depth is restrictive for root elongation, and is a diagnostic property defined in the World Reference Base (IUSS Working Group WRB, 2015) as a sharp increase of clay content within a depth-distance of 5 cm. The sharpness of changes over depth could not be assessed from the soil texture maps, because the size of the depth intervals increases with depth and gets too large at depth. Instead we defined an absolute increase from one interval to another, of the content of either clay (f.Clay) or sand (f.Sand), as indicative for the abruptness of textural change and set a mild, root permissive, threshold value which is valid over an assumed minimal distance of 15 cm.
4. *Cementation (induration)* of the soil is restrictive to root elongation when soil pores are filled by minerals that accumulate, either relatively or absolutely, and then precipitate and harden upon drying. Oxides of iron, aluminium and silica may cause induration in the form of a (petro-) plinthic, gibbsic or duric horizon. Associated data, required for mapping, were not available from the soil profiles datasets and rules were therefore not developed. Excessive contents in the soil of carbonates and sulphates, most commonly associated with calcium or magnesium, also causes soil to cement and the associated data were available. A content of CaCO3 exceeding 150 g/kg is a criterion to identify a calcic horizon which becomes petrocalcic when hardened, while a CaSO4 content exceeding 50 g/kg becomes petrogypsic when hardened (IUSS Working Group WRB, 2015). Further parameterisation was based on Landon (1991) and Sys et al. (1993).
5. *Acidity* restricts root development due to the acidity itself but also due to associated toxicities and nutrient deficiencies. A rule was parameterised through soil pH as measured in a soil-water suspension (pH-H_2_O), and as mapped by Hengl et al. (2015b), on the basis of Landon (1991), Hazelton and Murphy (2007), Sys et al. (1993), Sanchez et al., 1982, Sanchez et al., 2003, Brenes and Pearson (1973) and Kiniry et al. (1983). Little disagreement exists about 5.5 as the critical value for pH-H_2_O below which rootability is suboptimal (RI < 100%) but the lower limit (RI = 0%) is less well documented.
6. *Alkalinity* restricts rooting for several reasons and a rule was parameterised through pH-H_2_O based on the same literature consulted for acidity. Here again there is little disagreement about the critical value below which rootability is suboptimal, but there is little information about the lower limit. Hence, the thresholds as reported by Mulders et al. (2001) were used.
7. *Salinity* hinders root and crop growth, not only by toxicity effects or unbalanced nutrient uptake but also by increasing the osmotic pressure with negative impact on soil water availability and root turgor. A rule was parameterised based on FAO (1988), Sys et al. (1993), Sanchez et al. (2003), Kiniry et al. (1983) and Landon (1991) who report the impact of salinity on maize yield potential, with salinity expressed by electric conductivity as measured in a saturated paste (ECe) and water (ECw). However, because only scarce soil profile data were available on ECe, and none on ECw, maps of EC (electrical conductivity measured in an unsaturated extract) were produced using soil data (for over 17,000 profiles and 47,000 layers) queried for EC and excluding those for ECe. Only limited information was available on the effect of EC on root performance and consequently the parameters for evaluating ECe were adapted based on the relationship between ECe and EC as elaborated by Landon (1991) and Hazelton and Murphy (2007).
8. *Sodicity* strongly affects the physical conditions of soil and particularly of clayey soil which tends to disperse, resulting in low porosity which impedes rooting. As for salinity and alkalinity, it also causes nutritional imbalances and toxicity. Rules for sodicity were parameterised referring to FAO (1988), Landon (1991), Sanchez et al. (2003), Sys et al. (1993) and were based on the exchangeable sodium content and the exchangeable sodium percentage (ESP) relative to CEC.
9. *Toxicity* is commonly induced by very high acidity or alkalinity which leads to increased contents (ppm) of aluminium, iron, manganese, zinc, copper, boron, sulphur and other elements (including micro-nutrients). Because we did not have data for all of these elements from a majority of the profiles in the soil datasets, beyond a depth of 50 cm, rules were developed only related to exchangeable aluminium (cmolc/kg), which is assumed equal to exchangeable acidity at pH-H_2_O below 5.5, and the exchangeable aluminium percentage relative to CEC, based on Sanchez et al. (2003), Landon (1991), Brenes and Pearson (1973) and Hazelton and Murphy (2007).
10. *Soil morphology* determines rootability to a large extent and in various ways including ways similar to above described soil factors. Soil rooting conditions can be evaluated from descriptive data and qualitative information on soil morphology as shown by Driessen et al. (1997). We tried to interpret soil observations on soil structure, consistency, porosity, compaction, cementation, mottling (aeration) and specific features such as slickensides and information such as horizon designation, diagnostic criteria for soil classification and the type of soil (Baruth et al., 2006).

For six depth intervals, using the soil property maps as input for the rules developed and parameterised, maps were produced of the RI's associated with each of the soil factors. These RI's were splined through the six depth intervals and for each soil factor evaluated relative to the threshold indices to provide a continuous estimate of the depths, and the corresponding soil layers, at which rootability is restricted beyond the threshold indices.

#### 2.5.3 Mapping the depth of soil to the shallowest restriction for rooting

The rootable depth is assumed to be the shallowest of the depths evaluated from the individual soil layers (in which rootability was restricted beyond the threshold index for one of the soil factors considered) compared with the shallowest of the depths evaluated from the soil profiles as a whole (depth to bedrock and depth of aeration) and the depth of soil maximally attainable by the crop under unconstrained adequate conditions. The process involves:

1. *Mapping the depth of soil to a soil layer inadequate for rooting, with rootability restricted beyond a threshold.* This depth is evaluated from the ten soil factors as described in the previous section.
2. *Mapping the depth of soil to bedrock.* The depth of soil, potentially accessible for rooting, is limited by the depth of soil to bedrock (R) or to an indurated metal hard pan (Cms). A map of the depth of soil to bedrock was produced by DSM interpolating legacy soil data, for the soil profile as a whole, available for approximately 4700 profiles only (including 3660 virtual profiles interpreted and georeferenced from legacy soil maps depicting presence of an iron pan at shallow depth). Soil layers, not designated as an R horizon, with a coarse fragments content exceeding 90 v% were also considered as bedrock which added data for another 770 profiles. The depth of observation, which was reported for all soil profiles including the AfSS profiles which only consider the upper 50 cm of soil, is not indicative of the depth to bedrock but of the minimum depth of soil at which bedrock does not occur. These so-called censored observations (26,277) were also used for mapping depth of soil according to as described by Shangguan et al. (2017). The map was produced and cross-validated, using the same procedures as described in Hengl et al. (2015b), with a maximum depth on the map fixed at 175 cm.
3. *Mapping the depth of aerated soil.* Soil rootability is limited by oxygen shortage or poor aeration in the soil profile (Bengough et al., 2005; de Willigen and van Noordwijk, 1987). The depth of aeration is determined by the rate of water being drained from the soil and by the associated depth and duration, during rainy periods, of the soil being wet or saturated. We derived this depth of aeration from the drainage class, a soil profile property commonly reported during soil survey and recorded for 13,700 profiles from the AfSP database. The drainage classes range from very poorly drained (1) to excessively drained (7) as defined by Soil Survey Division Staff (1993). The field observations are subjective but quite easy to make correctly except for intermediate situations where the soil is imperfectly to moderately well drained. The qualitative nature of the definitions required additional literature review to define rules to interpret the ordinal (1–7) drainage classes as a quantitative depth (cm) of aerated soil or ‘depth to oxygen shortage during a large part of the cropping season’ (FAO, 1976; Landon, 1991; Sys et al., 1993; Cornell University, 2010). This interpretation was largely based on expert judgement and therefor it felt justified to define a mild rule to avoid disproportional impact on the final result. For details and rationale behind this rule, see Leenaars et al. (2015). A map of the depth of aerated soil was derived from the map of drainage classes. This map was produced and cross-validated, as ordinal- rather than categorical classes, using similar procedures as used in Hengl et al. (2015b) and additional covariates including the groundwater table depth (Fan et al., 2013).
4. *Depth of soil maximally attainable by the crop under unconstrained adequate conditions (150 cm for maize).*

The depths evaluated adequate for rooting were compared and the shallowest of those depths was assessed to produce the map of rootable depth or RZD (in cm). We also produced a map of the soil factor which is restricting RZD and assessed, for each soil factor, the extent (area in km^2^), degree (depth in cm) and severity (volume in km^3^) that RZD is restricted.

The map of RZD could not be validated quantitatively because (proxy-) data on rootable depth were not made available. Instead, we validated the map by expert judgements involving a team of soil scientists and agronomists. Errors, inconsistencies and odd patterns obvious at coarse scale were verified by comparison with reference soil maps and by proofing of the associated input data, both soil profiles data and the primary soil property maps, and of the rules for evaluation. Where necessary and possible, an improved version of the primary soil property maps was produced and validated and a new version of the RZD map was derived. See Leenaars et al. (2015) for details. As a sensitivity analysis, we computed Pearson correlations to assess the degree to which each of the soil properties, defining the soil factors, contributes to the variance of RZD.

### 2.6 Mapping the RZ-PAWHC

Maps for each depth interval for PAWHC (v%) of the soil fine earth were combined with the depth interval specific maps of the SFEF (v%) and aggregated over RZD into a weighted average single value for RZ-PAWHC (mm). The map of RZ-PAWHC could not be validated quantitatively due to the lack of adequate data on RZD. Instead, the map was validated by expert judgements similarly as how the map of RZD was validated. See Leenaars et al. (2015) for specific details. The sensitivity of RZ-PAWHC for each of its three components, and each of the underlying primary soil properties, was assessed by Pearson correlation.

## 3 Results

### 3.1 Methodological framework

The workflow to produce the maps of RZ-PAWHC was implemented within the overarching methodological framework as provided by the Global Soil Information Facility which is accessible at cran.r-project.org/web/packages/GSIF/. The procedures used for this study are publicly available online as implemented in the GSIF package for R software (Hengl et al., 2015a). All soil data are publicly available according to the data policy of ISRIC as the World Data Centre for Soils, including input data (soil profiles data, except the AfSS dataset, and primary soil property maps), intermediate results (maps derived per depth interval) and final results (maps aggregated over rootable depth), all at 1 km resolution. These data are available at the ISRIC ftp-server (username = public; password = public) which is accessible from www.isric.org/projects/afsis-gyga-functional-soil-information-sub-saharan-africa-rz-pawhc-ssa.

### 3.2 Input data for identified soil properties

#### 3.2.1 Soil profiles data

Of the variables used to evaluate PAWHC, SFEF and RZD, bulk density has poorest coverage of the data for the individual soil profile layers and depth to bedrock of the data for the soil profiles as a whole (Table 1). Data distribution seems normal for bulk density, pH-H_2_O, sand, silt, clay and drainage class. These data show similar mean and median values and are situated more or less in the middle of the range between minimum and maximum values. Data distribution is somewhat skewed for exchangeable acidity, CEC and organic carbon and is highly skewed for coarse fragments, electric conductivity and exchangeable sodium.

**Table 1:** Summary overview of the soil profiles data and soil profiles layer data.

		n^a^ (AfSP)	n^a^ (AfSS)	Mean	St Dev	Median	Shift^e^	Min-max	
Bulk density	kg/dm^3^	9291	0	1.38	0.23	1.40	−1.8	0.16–2.27	
Electric conductivity	dS/m	32,763	18,055	0.78	11.1	0.05	1460	0–776	c
Exch. sodium	cmolc/kg	46,225	1414	1.1	4.45	0.13	746	0–200	c
Coarse fragments	m^3^/100 m^3^	47,484	0	19.7	31.5	1.0	1870	0–100	
Drainage class^b^	–	13,704	0	4.3	1.25	5.0	−14	1–7	
Depth to bedrock^b^	cm	1047	0	79	76	70	12.8	0–1700	
Depth to bedrock^b^	cm	4708	0	33	44	30	10	0–1700	d
Censored depth to bedrock^b^	cm	16,677	9600	99	64	90	10	0–2000	
Organic carbon	g/kg	45,956	18,055	8.9	15.3	5.0	98	0–570	c
pH-H_2_O	–	54,867	18,055	6.2	1.2	6.0	3.3	2.1–11.3	c
Sand	g/100 g	58,322	1408	54	25	55	−1.8	0–100	c
Silt	g/100 g	58,318	0	16	13	13	23.1	0–100	
Clay	g/100 g	58,321	0	30	20	27.4	9.5	0–97	
CEC	cmolc/kg	52,886	0	14.4	15.1	8.3	73.5	0–179	
Exch. aluminium	cmolc/kg	26,791	0	0.7	2.3	0	–	0–76.7	

a Number of observations (either soil profiles or soil profile layers depending on the property).

b Soil properties observed and reported for the soil profile as a whole.

c Summary statistics from the AfSP database.

d Summary statistics from the AfSP database plus virtual profiles derived from legacy soil maps.

e Deviation (shift) of mean from median, relative (%) to median, indicative for skewness.

#### 3.2.2 Soil property maps

The soil property maps produced, including those reported by Hengl et al. (2015b), have an accuracy and statistics as summarised in Table 2. The summary statistics apply to the weighted averages of the depth intervals over the top 150 cm of soil, except for drainage class and depth to bedrock which apply to the soil profile as a whole. Not produced and not included in Table 2 are maps of calcium carbonate and gypsum content due to limited data availability and maps of soil morphologic properties due to the insufficiently consistent nature of the corresponding soil profile observation data.

**Table 2:** Summary overview of the accuracy of the soil property maps.

	Variance explained %	RMSE	Mean	St Dev	Median	Shift^a^	Min-max	Min-max 99%
Bulk density	70.3	0.13	1.45	0.12	1.45	0.3	0.74–1.99	1.15–1.80
Electric conductivity	60.7	8.09	5.9	25.8	0.5	1060	0–573	0–40
Exchangeable sodium	53.6	2.98	1.5	2.5	0.4	233	0–180	0–15
Coarse fragments	20.3	18.4	17.1	9.8	16.1	8.8	0–85	0–50
Drainage class	28.3	1.05	4.5	1.5	5.0	−10.3	1–7	1–7
Depth to bedrock	–	–	142	36	153	−7.3	6–175	10–175
Organic carbon	61.3	10.6	5.1	4.3	3.8	45.9	0–162	0.9–42
pH-H_2_O	66.9	0.67	6.4	1.1	6.2	3.5	4.2–10.6	4.4–8.7
Sand	61.1	15.9	51.9	13.6	50.7	2.3	6–97	7–94
Silt	56.1	8.3	16.8	5.9	17.1	2.0	0–50	1–47
Clay	52.4	13.7	31.3	9.6	31.4	−0.7	0–77	3–73
CEC	66.3	7.9	13.4	9.2	10.1	33.8	0–76	1.2–57
Exchangeable aluminium	77.3	1.3	0.9	1.1	0.3	165	0–23.4	0–6.4

a Deviation of mean from median, relative (%) to median.

The variance explained at the considered resolution is reasonable to good for most soil properties but is critically low for coarse fragments content and drainage class which is surprising given the amount of available soil data. The variance explained is not assessed for depth to bedrock. Root mean square errors (RMSE) are high, exceeding mean and median of predicted values, for electric conductivity, exchangeable sodium, exchangeable acidity and organic carbon content and, to a lesser extent, for coarse fragments content. High RMSE seem to correspond with large standard deviations in most cases and with skewness (shift) in the predictions. The skewness is large for exchangeable sodium and exchangeable acidity and very large for electric conductivity, though less than the skewness of the input soil profile data. The maps of coarse fragments do not reflect the skewness which was associated with the soil profile data.

**Fig. 3:**
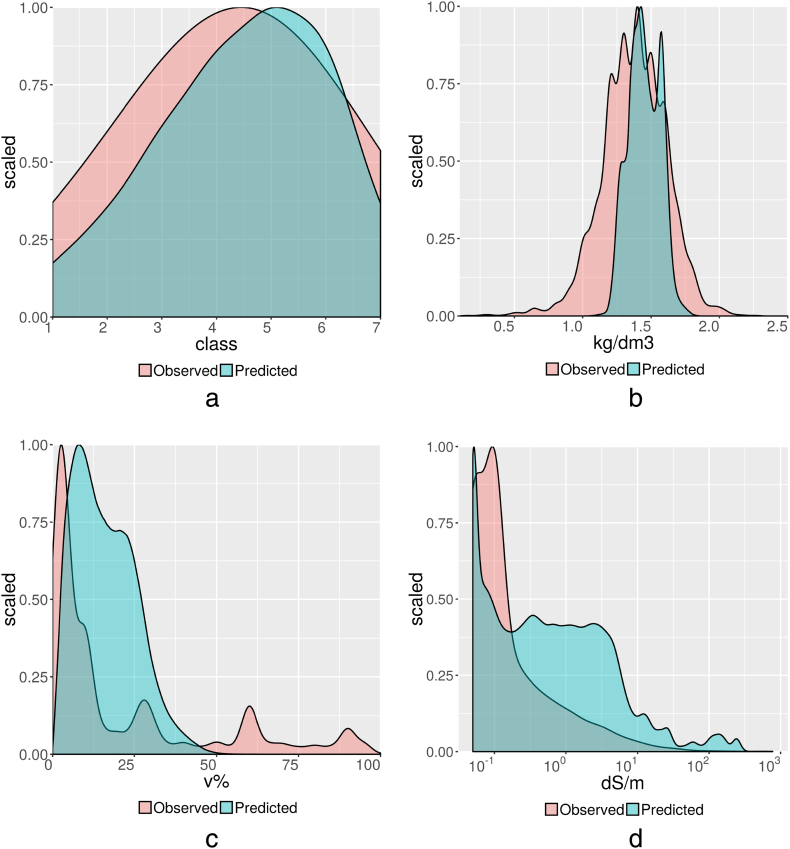
Scaled probability distributions of observed and predicted soil property values, shown at the back in pink and the front in blue, respectively, for a). drainage class and, aggregated over 150 cm depth, b). bulk density in kg/dm^3^, c). coarse fragments content in v% and d). electric conductivity on a logarithmic scale in dS/m. (For interpretation of the references to colour in this figure legend, the reader is referred to the web version of this article.)

The statistics of Table 1, Table 2 are described in more detail for selected soil properties (drainage, bulk density, coarse fragments content and electric conductivity) as illustrated in Fig. 3 by scaled probability distributions. The predicted drainage classes, aggregated from interpolated ordinal class values, are more or less normally distributed, spanning the full min-max range, comparable to the observations. The RMSE is fairly low but R^2^ is only 0.28, which could be particularly due to challenges associated with the intermediate classes (imperfect to moderately well drained).

Predicted values for bulk density have a similar distribution as the observed values, except for an average value which overestimates bulk density by 0.07 kg/dm^3^ and a much narrower min-max range. Modelling had an important smoothing effect which resulted in a loss of values at the higher and especially lower end which were observed but not predicted.

Observed values for coarse fragments content show a very skewed distribution with the average value exceeding the median by a factor of 1870%. (The peaks in Fig. 3c represent observed class values). The predicted average is near similar to the observed average but the median changed importantly from near nil to a value near similar to the much higher mean, which indicates a less skewed, more normal, distribution of the predicted values. At issue is whether this “smearing effect” is an improvement. 99% of the predictions is below 50 v%, which is a far too low maximum and requires improvement.

Reported data for electric conductivity are also distributed in a very skewed manner. The distribution of predicted values is less skewed. Predicted values are generally 10 times greater than measured values but the contrary occurs in relatively small, salty areas (of sizes not visible in Fig. 3d) where very high observed values (100's dS/m) have been underestimated. 99% of the predictions is below 40 dS/m. Again, modelling had an important smoothing effect, not only by narrowing the range of values but especially by “smearing” the values from a skewed to a more normal distribution. For exchangeable sodium and exchangeable aluminium similar remarks can be made as for electric conductivity although to a lesser extent.

For all soil properties, the range of predicted values is narrow compared to the range of measured values irrespective of the degree of skewness. This smoothing effect, caused by the applied DSM technique, is inherent to soil mapping, but is less at more local extents. A “smearing effect” occurs on very skewed data for properties with measured values which are generally very low but occasionally, in relatively small localised areas, very to excessively high. Results for these properties show large RMSE and a strong overrepresentation of mid-range values. See Leenaars et al. (2015) for more detailed statistics.

### 3.3 Maps of plant-available soil water holding capacity of the soil fine earth (PAWHC)

Results of the PTF, tested on the soil profile data and applied to the soil property maps, are summarised in Table 3 by measured and calculated VMC's, at saturation, field capacity (FC) and permanent wilting point (PWP), and PAWHC. The results at saturation (pF 0) are included here as part of the outcome of the PTF.

**Table 3:** Measured and calculated soil water retentions (VMC) and PAWHC (in v%).

	Profiles measured			Profiles calculated		Grids (150 cm) calculated				
	Mean	SD	Min-max	Mean	SD	Mean	SD	Median	Min-max	Min-max 99%
VMC at saturation	42.0	14.7	5.0–85	47.0		41.6	4.0	41.9	25–65	30–53
VMC at FC (2.0)	31.0	15.9	3.7–98	33.0						
VMC at FC (2.3)	26.0	15.5	2.3–98	29.1	±6	28.7	3.7	28.8	8–53	±13–44
VMC at FC (2.5)	21.1	14.0	1.0–98	28.0						
VMC at PWP	14.6	10.7	0.0–83	19.0		19.6	5.3	19.8	1–45	5–37
PAWHC 2.0	16.4	7.7		14.0	2.9					
PAWHC 2.3	11.4	7.1	0.1–56	10.1	2.7	9.1	1.6	9.0	0–20	3–14
PAWHC 2.5	6.5	4.9		7.9	2.5					

VMC calculated from the soil profiles data at pF 0, 2.0, 2.3, 2.5 and 4.2 overestimates measured VMC, in relative terms especially at higher tensions. The PTF underestimates PAWHC, with FC defined at pF 2.3, by one-ninth. PAWHC calculated for fine, medium and coarse textured soil profile layers, each with FC defined at pF 2.3, is on average 10.7, 11.1 and 8.6 v%, respectively, and this pattern corresponds with the measurements. Texture specific definition of FC results in a tendency of calculated PAWHC's contrary to what was anticipated and not conform the measurements. The definition of FC has very significant impact on PAWHC, both measured and calculated. PAWHC with FC defined at pF 2.0 exceeds PAWHC with FC defined at pF 2.5 with 9.9 v% measured and 6.1 v% calculated. The impact of the definition of FC on calculated PAWHC largely exceeds that of texture.

**Fig. 4:**
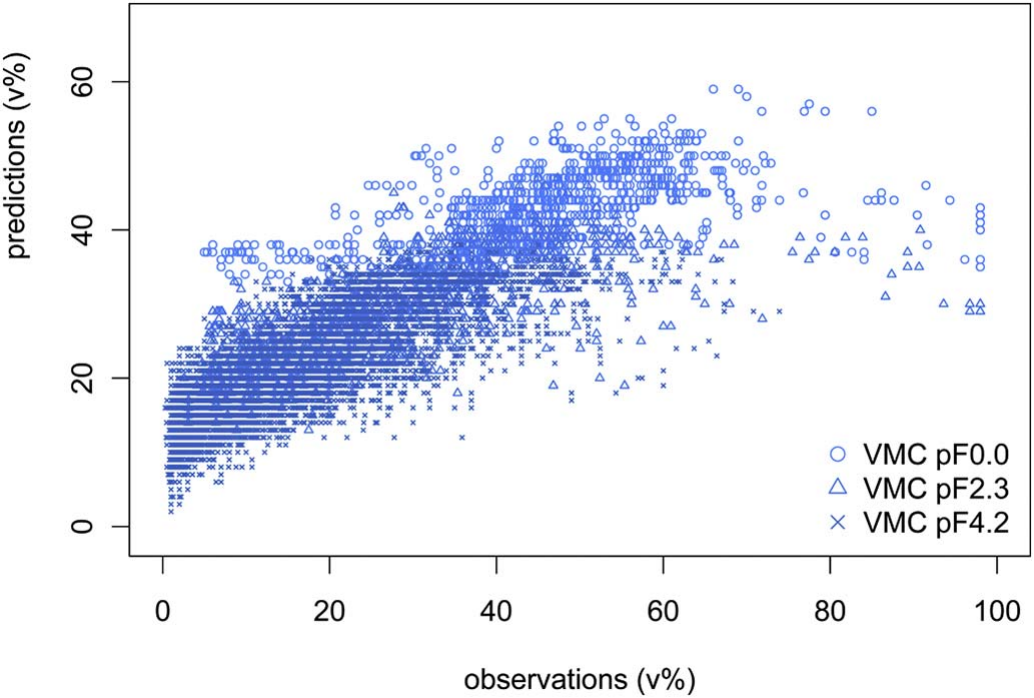
Predicted (mapped) versus observed VMC at saturation (pF 0), FC (pF 2.3) and PWP (pF 4.2).

The tabulated results of the PTF applied to the soil property maps represent the predictions for the six depth intervals aggregated over 150 cm of depth. The weighted mean VMC predicted at saturation is equivalent to the corresponding average of measured VMC, whereas the mean VMC predicted at FC (pF 2.3) and PWP overestimates the measured average with one-tenth and one-third, respectively. The min-max range of predicted VMC's is considerable but the range of measured VMC is nearly twice as large. The calculated and measured VMC's are normally distributed but the calculation of the maps had an important smoothing effect with underestimated high end values and especially overestimated low end values. This is to a certain extent due to the PTF and comparable to as tested on the soil profile data (which though mainly overestimated throughout the different tensions) and the fact that the underlying soil property maps do not depict any low and high end values either. The accuracy of the PTF combined with that of the soil property maps used as input data is reflected in Fig. 4 which shows, for six depth intervals, mapped (predicted = calculated) versus observed VMC (at saturation, FC and PWP). With *n* = 13,300, the variance explained (*R*^*2*^) is 0.72 and the *ME*, *MAE*, *RMSE* and *RMdSE* are 0.049 cm^3^/cm^3^, 0.084 cm^3^/cm^3^, 0.102 cm^3^/cm^3^ and 0.080 cm^3^/cm^3^, respectively. The accuracy of applying the PTF to the grids is comparable though slightly less than the accuracy reported by Wösten et al. (2013) who applied the PTF to the AfSP data with an *R*^*2*^ of 0.81 and a *RMSE* of 0.064 (cm^3^/cm^3^).

Derived and mapped PAWHC, in the soil fine earth, decreases from the first to the sixth depth interval from 9.6 to 9 v%. The weighted mean value mapped for PAWHC, with a similar median, is one-tenth below the mean value calculated from the profiles data which again is one-ninth below the mean measured value. The mapped predictions are normally distributed over a min-max range, which is very narrow compared to the range of measurements. In absolute terms, PAWHC derived for 150 cm deep soil is on average 137 mm and varies between 0 and 300 mm (mainly 45–210 mm). PAWHC is particularly sensitive to bulk density and silt content as suggested by Pearson correlations of −0.61 and 0.43, respectively (Table 6).

**Fig. 5:**
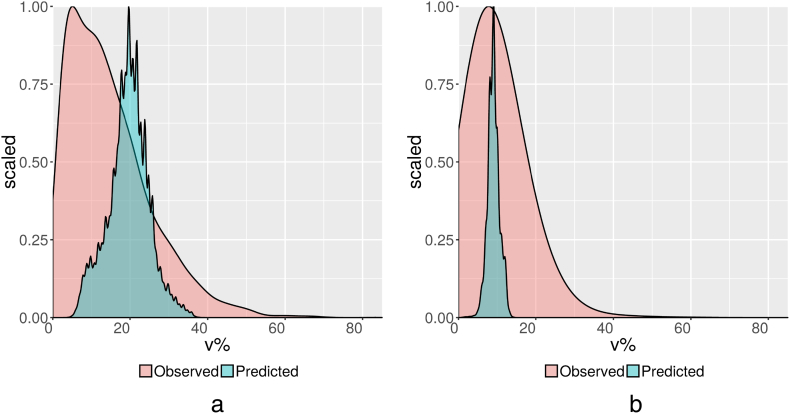
Scaled probability distributions of observed and mapped (predicted) a). VMC at PWP and b). PAWHC.

Fig. 5 visualises the scaled probability distributions of the observed and mapped (derived) VMC, at PWP, and PAWHC. PAWHC is reduced due to the overestimation of VMC at PWP.

**Fig. 6:**
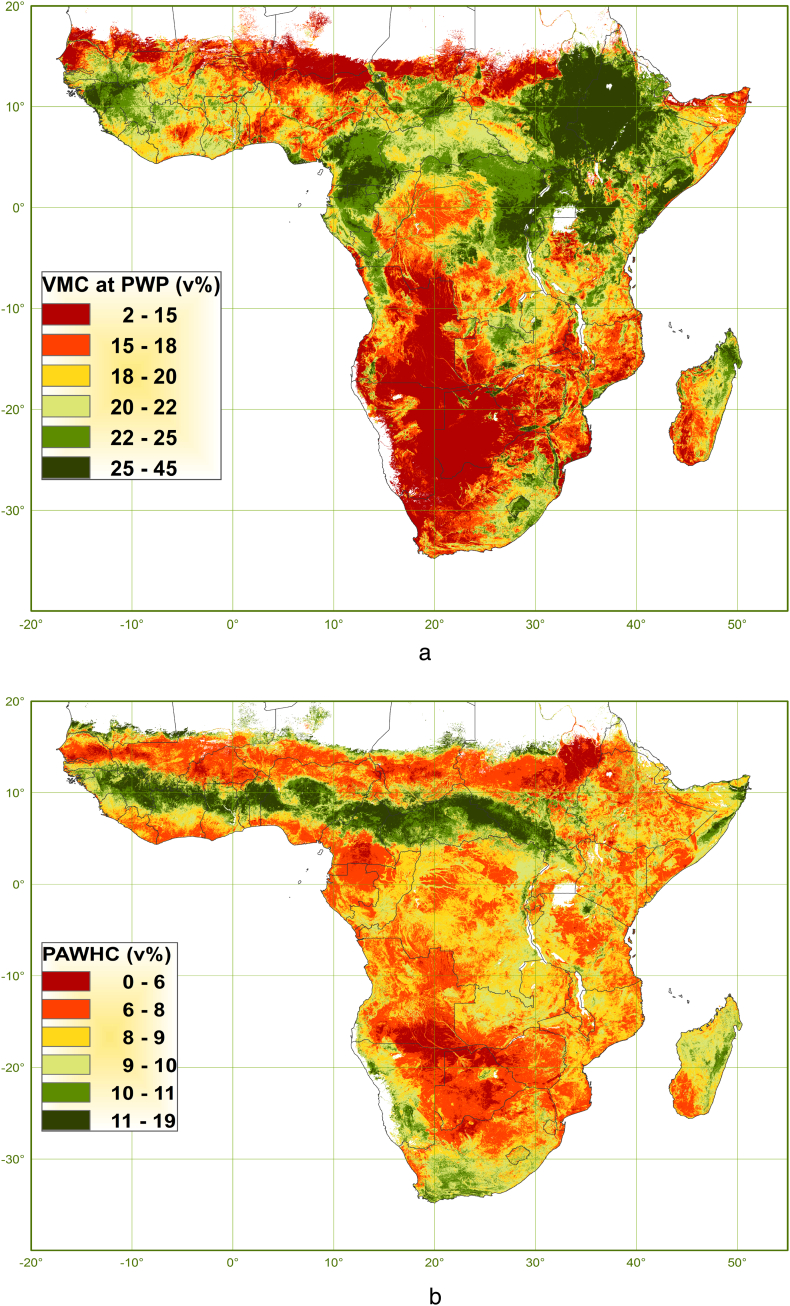
Maps of a). VMC at PWP and b). PAWHC in the soil fine earth.

Maps of VMC at PWP and of PAWHC are given in Fig. 6. Overall, the spatial patterns of predicted VMC show a large degree of variation, but the spatial variation of predicted PAWHC is limited. PAWHC is remarkably small in the Blue Nile in-land delta (Gezira), an area reputed for its extensive vertisols (smectite clays). PAWHC is also small throughout areas with arenosols (sandy soils) in west Southern and Western Africa. Surprisingly, relatively high PAWHC is predicted over the entire Guinean savannah zone stretching over west and central Africa. These larger PAWHC values are still only about half those of major grain-producing areas in temperate regions like the US Corn Belt and Argentine Pampas (www.yieldgap.org).

### 3.4 Maps of the soil fine earth volume as a fraction of the soil whole earth volume (SFEF)

**Fig. 7:**
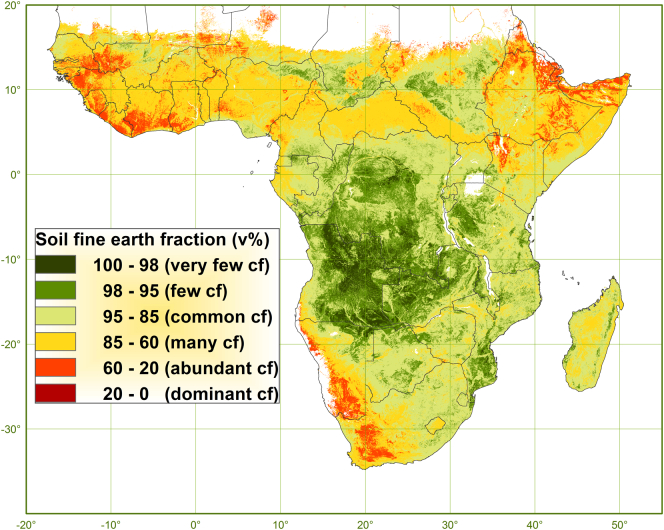
Map of the soil fine earth fraction (v%) depicted according to classes of coarse fragments (cf) content.

SFEF decreases from the 1st to 6th depth interval with about one-tenth from on average 90 to 80 v%. The mean weighted average over 150 cm of depth is 83 v% (±10) and varies between 15 and 100 v% of which 99% is between 50 and 100 v%. The effective PAWHC is reduced by the SFEF with one-sixth from on average 9.1 v% of the soil fine earth to 7.5 v% of the soil whole earth, which equals a reduction in absolute terms from 137 to 113 mm in 150 cm deep soil. See Fig. 7 for the map of SFEF.

### 3.5 Map of the rootable soil depth (RZD)

#### 3.5.1 Evaluation framework

The rules, parameters and criteria (thresholds), developed as part of the framework to evaluate and map rootability and rootable depth, are included as results in the next sections.

#### 3.5.2 Mapping rootability of soil layers relative to a threshold

The rules developed for evaluating rootability from the adequacy of soil factors, as parameterised by soil properties, are given in Table 4. The rootability index (RI) expresses the scalable adequacy for rooting at the corresponding soil property values. The four intervals between the five columns for RI represent the soil property ranges where rootability is either 100%, 100–20%, 20–0% or 0%. The unscaled rootability of either 100 or 0% is evaluated from the soil property threshold value at the threshold index of 20%. Four soil properties are expressed and evaluated as a function of another property as specified in the table footnote. Explained here is f.BD which evaluates bulk density relative to a critical, texture dependent, bulk density of 1.25 kg/dm^3^ for a pure clay soil and 1.60 kg/dm^3^ for a pure sand soil.

**Table 4:** Rules to evaluate the adequacy of soil factors to support rooting by maize, expressed by a rootability index (RI) and parameterised by soil properties.

Soil factor	Soil property	Variable	Unit	RI		**RI**^a^	RI	
				100-100%		**20%**	0–0%	
Porosity	Saturated moisture content	VMC-Sat	v%	100	40	**30**	27.5	0
Porosity	Bulk density fine earth, function of clay	f.BD	kg/dm^3^	<0	0	**0.24**	0.3	>0.3
Volume	Coarse fragments content	CrsVol	v%	0	80	**88**	90	100
Texture	Sand fraction	Sand	g/100 g	0	95	**99**	100	
Texture	Abrupt sand increase, over 2 intervals	f.Sand	∆ g/100 g	0	30	**50**	55	100
Texture	Abrupt clay increase, over 2 intervals	f.Clay	∆ g/100 g	0	30	**50**	55	100
Induration	Carbonate content	CaCO₃	g/kg	0	150	**400**	450	1000
Induration	Gypsum content	CaSO₄	g/kg	0	50	**300**	350	1000
Acidity	pH-H_2_O, low	pH-H_2_O	–	12	5.5	**4**	3.63	1
Alkalinity	pH-H_2_O, high	pH-H_2_O	–	1	7.8	**8.8**	9.05	12
Salinity	Electric conductivity, unsaturated	EC	dS/m	0	1.5	**5.7**	6.75	>6.75
Sodicity	Exchangeable sodium (+)	ExchNa	cmolc/kg	0	1	**4.2**	5	>5
Toxicity	Exchangeable aluminium (3+)	ExchAl	cmolc/kg	0	2.5	**5.7**	6.5	>6.5
Toxicity	Exchangeable aluminium saturation, CEC	f.ExchAl	%	0	35	**75**	85	100

f.BD = BD − (1.6 − (0.0035 × [clay])), f.Sand = [sand layer n] − [sand layer n − 1], f.ExchAl = [exchAl] × 100 / [CEC].

The soil property values associated with the threshold rootability index are indicated in bold.

a RI = 20% is the threshold rootability index. The associated soil property value is the threshold value.

Rootability of the soil depth intervals, considering each of the soil factors except for induration, is on average indexed at 71% (±17). Porosity is most frequently indexed as most limiting for rooting in any of the depth intervals, not necessarily beyond the threshold index, followed by sodicity, acidity and toxicity. Volume (coarse fragments) and salinity are never, and textural adequacy near never, indexed as most limiting to rootability. Sodicity, for those depth intervals where sodicity is evaluated most limiting, shows an average RI of 47% (±34), which is the lowest of all soil factors (except for sand content which is most limiting on only 1000 km^2^), and is the only factor for which the lower standard deviation goes beyond the threshold index.

The rules developed to assess the depth to a soil layer with a root-restrictive soil factor, by evaluating the rootability indices for each soil layer separately, apparently have little impact, in terms of spatial extent, on the estimated RZD. This is not necessarily due to the rules being too mild (root permissive) but more likely due to the mild threshold indices (at 20%). More stringent threshold indices, for e.g. acidity set at 30%, would result in more soil layers evaluated as root restrictive. It is also likely that the underlying soil property maps lack values that fall beyond the threshold values due to the smoothing effect of mapping which narrows the range of predictions relative to the range of values provided by the actual soil profiles data.

#### 3.5.3 Mapping the depth of soil to the shallowest restriction for rooting in the soil profile

The depth of soil to a soil layer inadequate for rooting is mapped from the results of the former section. The depth of soil to bedrock is mapped as described in section 3.1 and the map of the depth of aeration is derived from the drainage class map. The depth of aerated soil associated with drainage class 1 to 7 is 10, 40, 75, 115, 160, 210 and 265 cm, respectively. This corresponds with 2.5 x^2^ + 22.5 x − 15, with x = the ordinal drainage class (1–7).

**Fig. 8:**
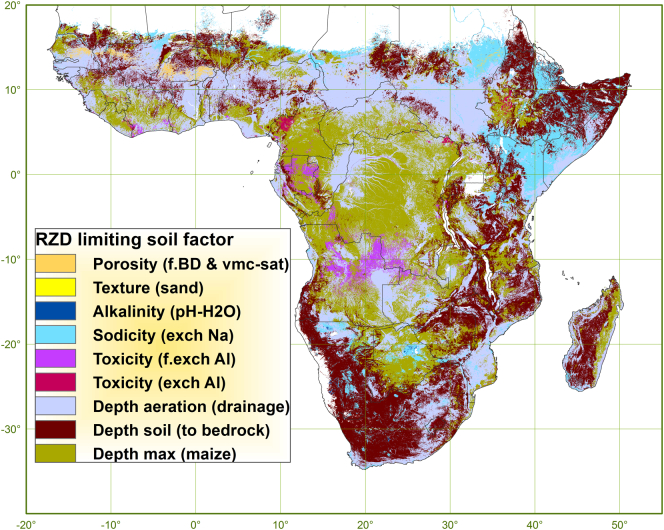
Map of soil factors limiting root zone depth.

Fig. 8 shows a map of the soil factors, with underlying soil properties, which are limiting RZD and which are either the depth of soil to a soil layer inadequate for rooting (with rootability restricted beyond a threshold), the depth of soil (to bedrock), the depth of aerated soil (to oxygen shortage) or the maximum rooting depth of maize (150 cm).

RZD is mapped as not restricted by soil conditions in 25% of SSA, especially in the humid tropics but also in semi-arid southern Africa (Angola, Botswana) and patches in the Sahel (Niger). In contrast, there are large regions where RZD is restricted by limited depth of aeration (36%); severely in much of the depression areas and wetlands with associated heavy clay soils as from lake Chad to South Sudan and, less severely but over very large extents, in areas occupying intermediate landscape positions where soils are imperfectly- to moderately well drained, often associated with pseudo-gley and plinthite, like in the savannahs stretching from Senegal to Nigeria and in Mozambique and also in the sandy gley soils along the Congo river. RZD is restricted by depth of soil to bedrock in 26% of SSA in large parts of the highlands of eastern and southern Africa, the petro-plinthite areas in western Africa and the areas with calcium-cemented soils in the far south-west and far north-east of Africa. In a relatively smaller area (13% of SSA) RZD is restricted due to other soil factors, including sodicity, toxicity, porosity, and alkalinity. Sodicity restricting RZD occurs in depression areas in arid zones such as along the border of the Sahara, the inland deltas in Mali, Namibia and Botswana and especially in the arid lowlands in- and bordering Ethiopia including the solonetz areas of Somalia and northern Kenya, the vertisol area of the Gezira in Sudan and the Ethiopian Danakil. Toxicity related to exchangeable aluminium (acidity) restricts RZD in the south of the Democratic Republic of Congo and the north of Angola, Gabon and the wetter parts of Cameroon, Ghana, Ivory Coast and Ethiopia. Porosity restricts RZD in parts of the Sahel over a narrow stretch from Senegal to Burkina Faso. RZD is restricted by alkalinity and by texture (excessive sandiness) in extremely small areas only. The other soil factors evaluated for their adequacy to support rooting are not identified as root restrictive beyond the rootability threshold based on the dataset used in this assessment.

**Fig. 9:**
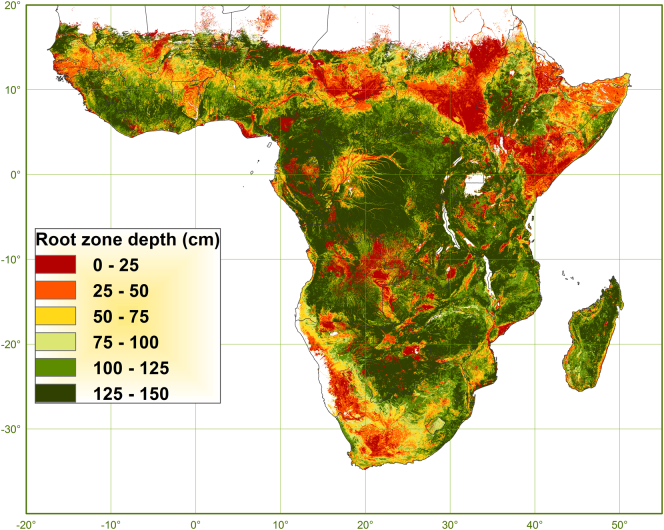
Map of rootable soil depth for maize (cm).

RZD is mapped for SSA as shown in Fig. 9 and is on average 96 cm (±49) with a range between 1 and 150 cm. The median is 20 cm deeper with a value of 115 cm. These figures are comparable with observed rooting depths as recorded in the AfSP database (*n* = 3970) with an average and median of 94 cm (±45) and 100 cm, respectively, in a range between 0 and 400 cm. This comparison is only a casual observation because the AfSP data reported for rooting depth are not specific for maize and not at a given moment in the growing period. The RZD map was not validated quantitatively because RZD data were not available.

An overview of both the extent (area in 1000 km^2^) and the degree (depth in cm) to which each soil factor is limiting RZD is given in Table 5. In terms of extent, RZD is generally more limited by soil factors evaluated for the soil profile as a whole, such as depth of aeration and depth to bedrock, than by soil factors evaluated for the individual soil profile layers separately, such as sodicity and toxicity. In terms of degree, or the magnitude of decrease in rootable depth, the contrary is true.

**Table 5:** Areas (1000 km^2^) and depths (cm) of soil factors restricting RZD.

Soil factor	Variable	RZD							RZD	RZD	
		0–5 cm	5–15	15–30	30–60	60–100	100–150	150	0–150 cm	avg	sd
		1000 km^2^							1000 km^2^	cm	cm
Porosity	VMC-Sat	<0.1	<0.1	0	<0.1	0	<0.1	0	<0.1	12	23
Porosity	f.BD	11.7	4.7	9.7	44.9	<0.1	3.9	0	123.7	51	27
Volume	CrsVol	0	<0	0	0	0	0	0	0	–	–
Texture	Sand	0	0.1	0	0	<0.1	0.1	0	0.1	131	25
Texture	f.Sand	0	0	0	0	0	0	0	0	–	–
Texture	f.Clay	0	0	0	0	0	0	0	0	–	–
Acidity	pH-H_2_O	0	0	0	0	0	0	0	0	–	–
Alkalinity	pH-H_2_O	6.8	2.2	4.7	2.9	0.7	0.2	0	17.5	19	22
Salinity	EC	0	0	0	0	0	0	0	0	–	–
Sodicity	ExchNa	977.0	27.7	279.8	575.9	98.6	27.3	0	1986.2	21	24
Toxicity	ExchAl	86.1	0.1	0.2	0.4	0.6	0.4	0	87.7	2	10
Toxicity	f.ExchAl	5.5	35.5	255.5	79.1	14.9	1.8	0	392.3	27	14
Depth layer		1087	70.2	549.8	703.1	163.6	33.8	0	2607.7	23	22
Depth aeration	f.Drain	0	760	0	1290	2313	2989.5	0	7352.3	78	36
Depth soil	Rock	0	2.3	147.9	627.5	1136.8	3327.7	0	5242.1	105	35
Depth max	150 cm	0	0	0	0	0	0	5169.1	5169.1	150	0
Total (1000 km^2^)		1087.1	832.2	697.7	2620.7	3613.6	6350.9	5169.1	20,371.2	96	49

The evaluated area of a size of 20.4 M km^2^ represents a soil volume potentially rootable by maize of 30,600 km^3^ (i.e. RZD not restricted by soil conditions). The rootable soil volume (of the SFEF, not considering coarse fragments content) is reduced by 10,527 km^3^ (one third) due to root-restrictive soil conditions, of which 4785 km^3^ are due to limited depth of aeration and 2517 km^3^ due to sodicity. Depth to bedrock reduces the rootable soil volume by 2478 km^3^, aluminium toxicity by 606 km^3^, porosity by 118 km^3^, alkalinity by 23 km^3^ and the other factors by practically 0 km^3^.

Some of the soil property maps underpinning the soil factors found to be most restrictive to RZD, either in extent or degree, are of critically low accuracy. The accuracy of the drainage class map, which underlies the evaluation of depth of aeration, seems to be particularly limited due to the challenges associated with predicting intermediate situations (imperfectly to moderately well drained, restricting RZD to 75 and 115 cm, respectively), which are relatively difficult to correctly describe in de field and generally located at intermediate landscape positions occupying very large areas. The accuracy of the exchangeable sodium map, which underlies the evaluation of sodicity and which restricts rootability at relatively shallow depth in some large lowland areas, seems to be compromised due to the skewed soil profiles data of which, moreover, a large portion is poorly inferred from spectroscopic data. The sensitivity of RZD for particularly these two soil properties is confirmed by the Pearson correlations (see Table 6). RZD proves also sensitive to pH-H_2_O and in a lesser extent to CEC which is particularly due to the covariance with sodium content.

**Table 6:** Pearson correlations.

	PAWHC	SFEF	RZD	RZ_PAWHC
PAWHC	1	–	–	0.21
SFEF	–	1	–	0.27
RZD	–	–	1	0.95
Bulk density	−0.61	–	−0.22	−0.38
Electric conductivity	–	–	−0.14	−0.12
Exch. sodium	–	–	−0.57	−0.57
Coarse fragments	–	−1	−0.22	−0.27
Drainage class	–	–	0.57	0.5
Depth to bedrock	–	–	0.34	0.35
Organic carbon	0.18	–	0.1	0.15
pH-H_2_O	−0.05	–	−0.51	−0.55
Sand	−0.22	–	0.14	0.04
Silt	0.43	–	−0.3	−0.18
Clay	0.05	–	−0.01	0.05
CEC	−0.11	–	−0.44	−0.45
Exch. aluminium	–	–	0.19	0.24

### 3.6 Map of the root zone plant-available water holding capacity (RZ-PAWHC)

Aggregated over RZD, and expressed in relative terms, average PAWHC of the soil fine earth fraction is 8.9 v% (±1.6), in a range between 0 and 19 v%. The average soil fine earth fraction is 86 v% (±9), ranging between 17 and 100 v%. The effective PAWHC in the RZD is on average 7.7 v% (±1.4) and ranges between 0 and 16 v% (mainly 3–11 v%). The spatial pattern of the effective PAWHC in the RZD corresponds to a certain extent with that of the soil water holding capacity suggested by Jones et al. (2013). Important differences occur as well though. The capacity depicted by Jones et al. (2013) ranges more widely from below 1.5 v% in Guinea to above 15 v% in large parts of Central and East Africa and is relatively low (5 v%) for the Guinea/Sudan savannah zone stretching over west and north-central Africa where we derived relatively high effective PAWHC.

**Fig. 10:**
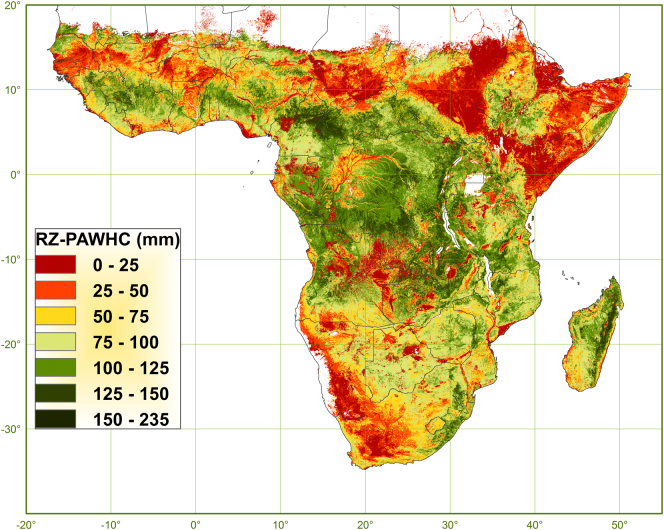
Map of the root zone plant-available water holding capacity (mm).

Fig. 10 shows the map of RZ-PAWHC expressed in absolute terms (in mm). Derived values for RZ-PAWHC range between 0 and 235 mm of which 99% is between 0 and 145 mm. The mean value is 74 mm (±39) with an almost similar median value. The spatial pattern of RZ-PAWHC is very comparable to that of RZD. As a general statement, RZ-PAWHC is in terms of extent (km^2^) more limited due to RZD limited by soil factors evaluated for the soil profile as a whole, such as depth of aeration and depth to bedrock (12.6 M km^2^), than by soil factors evaluated for the individual soil profile layers, such as sodicity and toxicity (2.6 M km^2^). In terms of degree (mm), or the magnitude of decrease in RZ-PAWHC, the contrary is true with RZ-PAWHC limited to on average 68 mm (±30) due to RZD limited by a restriction evaluated for the soil profile as a whole (depth of aeration and depth to bedrock) and to on average 15 mm (±16) due to RZD limited by a restriction evaluated for the individual soil profile layers (sodicity, toxicity).

Summarising, RZ-PAWHC would be 150 mm in soil without any limitations (with a default PAWHC set at 10 v%), 137 mm when limited by PAWHC, 125 mm when limited by SFEF and 96 mm when limited by RZD. RZ-PAWHC is thus limited by PAWHC with, in relative terms, 9% (±16), by SFEF with 17% (±39) and by RZD with 35% (±26), which suggests that RZ-PAWHC is most limited due to restricted rootability. Accumulatively, RZ-PAWHC is limited with 9% from 150 to 137 mm due to limited PAWHC, with 25% to 113 mm due to both limited PAWHC and SFEF and with 51% to 74 mm due to limited PAWHC, SFEF and RZD.

Table 6 gives the Pearson correlation coefficients between the map of RZ-PAWHC and the maps of PAWHC, SFEF and RZD and between each of these with the maps of the underlying soil properties. The correlations confirm that RZ-PAWHC is predominantly defined by RZD and most sensitive to those soil properties for which RZD is most sensitive (sodium content, drainage class and pH-H_2_O).

## 4 Discussion

This work resulted in spatially explicit and quantitative maps of the rootable depth and the RZ-PAWHC of the sub-Saharan African soil. While the accuracy of these maps could not be validated due to absent data on rootability or rooting, and is likely limited by the accuracy of the primary soil property maps, the framework used to create the maps is robust and the maps, either for SSA or specific areas of interest, can be updated as additional soil data become available.

The maps of RZD and RZ-PAWHC were derived from soil property maps which were produced by interpolation of soil profiles data using high resolution covariates. Value was added by combining legacy soil data (AfSP) with newly collected soil data (AfSS) to produce the soil property maps (Leenaars et al., 2014b; Hengl et al., 2015b, Hengl et al., 2017a). Particularly relevant are the differences in recorded soil properties and covered depths of soil. The AfSS dataset lacks depth to bedrock, drainage class and water retention, coarse fragments content and bulk density while other properties are recorded to a depth of 50 cm only. The AfSP dataset includes these properties and data are recorded for a depth to on average 125 cm, allowing to produce maps beyond 50 cm, which was needed for estimating and mapping RZD and RZ-PAWHC.

The accuracy of the primary soil property maps, which was assessed by cross-validation, is for most properties satisfactory but needs to be further improved for drainage class and coarse fragments content and preferably also for electric conductivity and exchangeable sodium. In all cases, high- and low end values measured and recorded in the soil profiles datasets appear to be not sufficiently well captured and represented by the spatial predictions. This “regression to the mean” or smoothing effect is quite common in any prediction method and deserves attention in future updates. The solution would be to use stochastic simulations (Webster and Oliver, 2007) avoiding smoothing and reproducing the statistics of the conditioning data. However, this would increase computing time dramatically and was beyond the scope of this study. Particularly difficult to map accurately were soil properties with limited data availability (depth to bedrock, bulk density), with low accuracy of source data (drainage and coarse fragments content derived from class values observed in the field and sodium and electric conductivity poorly inferred from spectroscopic data) and with skewed data distribution (coarse fragments, sodium, electric conductivity). To enhance model performance, particular attention is to be paid to obtain spatial covariates that are likely relevant for predicting these soil properties based on pedologic knowledge about soil forming processes.

Uncertainties in these maps will propagate through the subsequent analysis that computes the maps of PAWHC, SFEF and RZD and therewith of RZ-PAWHC. In addition each step of the analysis introduces additional uncertainties resulting from the uncertainties associated with the rules used for the inferences, including the assumptions, parameterisations and criteria. Thus, it is highly desirable to quantify these uncertainties and trace the propagation into the final product. However, analysing the propagation of uncertainties is computationally very intensive and requires that all sources of uncertainty are quantified by probability distributions, including spatial and cross-correlations of uncertain inputs (Heuvelink, 1998, Heuvelink, 2014). Such analysis was beyond the scope of this work but we intend to include mapping of the uncertainties and propagated errors in a next round of map updating. That work may benefit from the possibilities offered by the soil inference system as put forward by McBratney et al. (2002) and implemented by Morris et al. (2012).

The estimation of water retention, by applying a PTF to the profiles data and the soil property maps, appeared to be reasonably accurate, also when related to different texture classes. A slight overestimate was obtained, especially at higher tensions and PAWHC is therefore slightly underestimated generally. This can be corrected, for instance by adjusting the PTF parameters (Hodnett and Tomasella, 2002). PAWHC proved very sensitive to the definition of FC and would had been approximately four-tenth higher with FC defined at pF 2.0 and two-tenth lower with FC defined according to the GlobalSoilMap specifications (Arrouays et al., 2014) at pF 2.5 The forms of the predicted retention curves vary to a limited extent only and thus lead to values for PAWHC with little variation. The little variation is also due to the narrow range predicted in the underlying soil property maps, lacking low- and/or high end values. While Heuvelink and Pebesma (1999) recommend to first interpolate and then calculate, rather than vice versa, it may be worthwhile trying to first apply the PTF to the profiles data, which we did, and then interpolate the calculated data on water retention. This may result in a wider range of PAWHC. The feasibility of such approach is currently limited by the availability of sufficient data on bulk density and it required additional PTF's to generate those data. Pearson correlation showed that bulk density is key to estimate PAWHC and errors in the first round map of bulk density contributed to strange patterns, as detected by Han et al. (2015), in the first round map of PAWHC.

We estimated the RZD as determined by four major depth parameters, i.e. (a) depth of soil to the shallowest layer with a soil factor restricting rootability beyond the established threshold index, (b) depth of soil to bedrock, (c) depth of aerated soil, and (d) genetic root depth potential of maize. The last three depth parameters, and especially depth of aerated soil, appear to dominate the outcomes in terms of extent (area in km^2^), whereas the first, and especially depth to excessive sodicity, dominates in terms of degree (depth in cm). This makes the procedure rather sensitive to possible errors in the underlying maps of drainage class and exchangeable sodium content and the associated rules. The sensitivity of RZD for particularly these two soil properties is confirmed by Pearson correlations. However, the accuracy of the drainage class map is critically low and the sodium maps suffered from important smoothing. The low amount of variance explained by the drainage class map, despite the amount of data, could be due to challenges with the observation and prediction of intermediate classes on intermediate landscape positions, but also with the aggregation of 71 interpolated ordinal classes (1.0–7.0) into seven classes (1–7). The latter should be avoided in a next update. Further, it would be worthwhile trying to interpolate aerated depth of soil from depths of aerated soil as inferred directly from the soil profile data (first calculate, then interpolate). It would be most direct to use a map of the depth to groundwater (Fan et al., 2013) as the map of aerated depth of soil, if sufficiently accurate and precise. (It should be mentioned here that a limited depth of aeration, while limiting RZ-PAWHC, may well have positive effect on the supply of water into the root zone, due to possible capillary rise). The sodium maps generally overestimate the, skewed, measurements. Root restrictive sodium contents were predicted for all lowlands throughout SSA and we introduced additional covariates to “force” sodium out of the lowlands of the humid tropics, where measurements show low sodium contents, but this caused an increase of sodium in the arid regions at the other side of the spectrum. This black box effect seems a disadvantage relative to conventional soil type mapping which permits to allocate measured, possibly extreme, values to delineated soils. For a next update of the sodium map, it may be worthwhile to use covariates deducted from soil type maps and simplified into either presence or absence of sodic or natric characteristics.

The four major depth parameters in the framework are evaluated from twelve soil factors, identified to evaluate the adequacy of soil to support root growth, for which rules have been developed and parameterised based on sixteen soil properties. These rules are consistent and reliable, whereas a few, particularly the rule to evaluate the abruptness of textural change, deserve additional attention. The abruptness of textural change could be evaluated more consistently from textures given by a continuous depth function rather than by depth intervals which increase with depth. For the majority of rules, parameterisation also appears to be quite reliable. Parameterisation was mild in those cases that literature was too ambiguous. However, many of the, unscaled, thresholds that evaluate the rootability of soil depth intervals are relevant only for quite extreme soil property values, which do occur in the soil profiles datasets but, except for sodium, rarely or not on the maps. Consequently, several of such soil factors, e.g. acidity (pH-H_2_O), are nowhere on the map identified as root-restrictive. This is partly the result of, again, the smoothing effect that is inherent to mapping. Using stochastic simulation mentioned above, thresholds would be exceeded in some of the simulations, thus quantifying the probability of threshold exceedance and hence giving a measure of risk (Vann et al., 2002; Webster and Oliver, 2007). Not meeting the thresholds is also the result of the mildness, or root permissiveness, of the thresholds set for the various rootability indices. More stringent threshold indices, i.e. for acidity raised from 20 to 30%, would imply more stringent threshold values at which rootability is evaluated as restricted, i.e. at pH-H_2_O of 4.2 instead of 4.0. Instead of using thresholds, a scalable approach could be used in line with what was suggested by Driessen and Konijn (1992) or Kaufmann et al. (2009). Such would require the soil factors to be independent though.

RZD determines RZ-PAWHC to a much larger extent than PAWHC and SFEF. However, data availability did not permit to adequately validate rootability and RZD, and therefore RZ-PAWHC. Field observations on restricted auger depths, collected according to the land degradation surveillance network procedures (Vågen et al., 2010, Vågen et al., 2016), could serve as proxy for restricted rootable depths but data have not become available. In general, the accuracy of the RZ-PAWHC map is limited by the accuracy of the soil property maps from which the RZD map was derived. Besides, by using another DSM technique, these maps might be improved with additional soil profile data, either from existing data sources or newly collected from the field. Such data, in support to updating the current maps, should include the depth of soil (up to bedrock) and the depth of aeration in the soil (up to groundwater or inferred from drainage class) to preferably at least 100 cm depth and, for each of the soil profile horizons, the volumetric fraction of soil fine earth (coarse fragments), porosity (bulk density), texture (sand, silt, clay), cementation (CaCO3 and CaSO4), acidity and alkalinity (pH-H_2_O), salinity (EC), sodicity (exchangeable sodium and CEC) and toxicity (aluminium and others). Also relevant and sufficiently robust to map and parameterise, but not used in this study because of insufficient standardised data, are morphologic observations expressed simply as presence or absence of diagnostics like slickensides, abrupt textural change and highly compacted and/or cemented layers (e.g. duripan, iron pan). Much value would be added, also for local assessments, by indeed collecting these soil data together with data, per soil layer, on the actual presence or absence of roots (while specifying the species) which enables validation and fine-tuning of the rules to evaluate RZD.

The impact of the estimated RZD, and RZ-PAWHC, on crop yield potentials (Guilpart et al., 2017) and therewith the prognosis of whether SSA can feed itself is significant and leads to the conclusion that agricultural intensification alone may not be sufficient for reaching food security (van Ittersum et al., 2016). This conclusion has far-reaching consequences and justifies increasing efforts to better assess and map soil rootability and RZ-PAWHC in SSA to better target agronomic R&D interventions and better inform agricultural policy- and decision making.

## 5 Conclusions

This study produced the first map of rootable depth and RZ-PAWHC of the soil of SSA. The mean rootable depth (for maize as a reference crop) is 96 cm (±49 cm) and RZ-PAWHC is on average 74 mm (±39 mm) ranging from 0 to 235 mm (99% from 0 to 145 mm). RZD is by far the most important of the three components defining RZ-PAWHC (Pearson correlations with PAWHC, SFEF and RZD of 0.21, 0.27 and 0.95, respectively). RZD in its turn is from all soil properties most sensitive to drainage class and sodium content. Rootability is restricted at a depth of less than the genetically defined maximum RZD (150 cm) on three quarters of the total area of SSA and the total soil volume which is potentially rootable by maize is reduced by one third, due to root constraining factors as aeration, sodicity, bedrock, aluminium toxicity, and others. The accuracy of the RZD map could not be validated quantitatively, due to absent data on rootability and RZD, but is limited by the accuracy of the soil property maps from which the map was derived. Most of these soil property maps are smoothed compared to the observations (regression to the mean), especially for properties with skewed data. New, improved maps can be produced, within the operational framework here developed, upon the availability of additional soil data relevant to evaluate RZ-PAWHC over at least 100 cm depth and possibly using different DSM techniques such as stochastic simulation. Key in such update is to map the uncertainties associated with the soil property maps and to assess how the errors propagate into the RZ-PAWHC map. Adequate data on rootability, or rooting, are solicited to better validate the current assessment as a key step towards an increasingly accurate consolidated product, which is critically important for better targeting agronomic R&D interventions in SSA.

## Declaration of interest

Johan G.B. Leenaars, “Conflicts of interest: none”.

Lieven Claessens, “Conflicts of interest: none”.

Gerard B.M. Heuvelink, “Conflicts of interest: none”.

Tom Hengl, “Conflicts of interest: none”.

Maria Ruiperez González, “Conflicts of interest: none”.

Lenny G.J. van Bussel, “Conflicts of interest: none”.

Nicolas Guilpart, “Conflicts of interest: none”.

Haishun Yang, “Conflicts of interest: none”.

Kenneth G. Cassman, “Conflicts of interest: none”.

## References

[bb0005] Africa Soil Information Service (AfSIS) http://africasoils.net/.

[bb9000] Africa Soil Information Service (AfSIS) (2013). WorldClim 2013 Release. Center for International Earth Science Information Network (CIESIN).

[bb0010] Alexandratos N., Bruinsma J. (2012). World Agriculture towards 2030/2050: The 2012 Revision. ESA Working Paper No. 12-03.

[bb0015] Arrouays D., McBratney A.B., Minasny B., Hempel J.W., Heuvelink G.B.M., MacMillan R.A., Hartemink A.E., Lagacherie P., McKenzie N.J., Arrouays (2014). The GlobalSoilMap Project Specifications. GlobalSoilMap, basis of the global spatial soil information system.

[bb0025] Baruth B., Genovese G., Montanarella L. (2006). New Soil Information for the MARS Crop Yield Forecasting System.

[bb0030] Bengough A.G., Bransby M.F., Hans J., McKenna S.J., Toberts T.J., Valentine T.A. (2005). Root responses to soil physical conditions; growth dynamics from field to cell. J. Exp. Bot..

[bb0035] Boulet R., Leprun J.C. (1969). Carte pédologique de reconnaissance de la république de Haute-Volta: notices et cartes au 1/500 000.

[bb0040] Bouma J. (1989). Using soil survey data for quantitative land evaluation. Adv. Soil Sci..

[bb0045] Brenes E., Pearson R.W. (1973). Root responses of three gramineae species to soil acidity in an oxisol and an ultisol. Soil Sci..

[bb0050] Brooks R.H., Corey A.T. (1966). Properties of porous media affecting fluid flow. J. Irrig. Drain. Div. Am. Soc. Civil. Eng..

[bb0055] Cornell University (2010). Northeast Region Certified Crop Advisor (NRCCA), Study Resources. CA3: Drainage and Irrigation.

[bb0060] de Willigen P., van Noordwijk M. (1987). Roots, Plant Production and Nutrient Use Efficiency. PhD Thesis.

[bb0065] de Wit C.T. (1992). Resource use efficiency in agriculture. Agric. Syst..

[bb0070] Dercon S., Christiaensen L. (2007). Consumption risk, technology adoption and poverty traps: evidence from Ethiopia. World Bank Policy Research Working Paper.

[bb0075] DRC (1967). Soil survey of the southwest region. A Report Prepared for the Government of the Republic of Ivory Coast. Technical Appendix.

[bb0080] Driessen P.M., Konijn N.T. (1992). Land-Use Systems Analysis.

[bb0085] Driessen P.M., Ihle M.W., Leenaars J.G.B. (1997). Land Suitability Assessment for Selected Land-Use Systems in the Sanmatenga-North Area, Burkina Faso.

[bb0090] Fan Y., Li H., Miguez-Macho G. (2013). Global patterns of groundwater table depth. Science.

[bb0095] FAO (1976). A framework for land evaluation. FAO Soils Bulletin 32.

[bb0100] FAO (1978). Report on the agro-ecological zones project. Methodology and Results for Africa. World Soil Resources Report 48.

[bb0105] FAO (1988). Salt-affected soils and their management. FAO Soils Bulletin 39.

[bb0110] FAO (2006). Guidelines for Soil Description.

[bb0115] Garnett T., Godfray C. (2012). Sustainable intensification in agriculture. Navigating a course through competing food system priorities. Food Climate Research Network and the Oxford Martin Programme on the Future of Food.

[bb0120] Gijsman A.J., Thornton P.K., Hoogenboom G. (2007). Using the WISE database to parameterize soil inputs for crop simulation models. Comput. Electron. Agric..

[bb0125] Global Yield Gap and water productivity Atlas (GYGA) http://www.yieldgap.org.

[bb0130] GlobalSoilMap (2015). Specifications Tiered GlobalSoilMap Products. Release 2.4. http://www.globalsoilmap.net/specifications.

[bb0135] Grassini P.L.G., Van Bussel J., Van Wart J., Wolf L., Claessens H., Yang H., Boogaard H., De Groot M.K., Ittersum Van, Cassman K.G. (2015). How good is good enough? Data requirements for reliable yield-gap analysis. Field Crop Res..

[bb0140] Guilpart N., Grassini P., van Wart J., Yang H., van Ittersum M.K., van Bussel L.G.J., Wolf J., Claessens L., Leenaars J.G.B., Cassman K.G. (2017). Rooting for food security in Sub-Saharan Africa. Environ. Res. Lett..

[bb0145] Han E., Ines A., Koo J. (2015). Global High-Resolution Soil Profile Database for Crop Modeling Applications, Working Paper.

[bb0150] Hazelton P., Murphy B. (2007). Interpreting Soil Test Results: What Do all those Numbers Mean? CSIRO.

[bb0155] Hendriks C.M.J., Stoorvogel J.J., Claessens L. (2016). Exploring the challenges with soil data in regional land use analysis. Agric. Syst..

[bb0160] Hengl T., Heuvelink G.B.M., Stein A. (2004). A generic framework for spatial prediction of soil properties based on regression-kriging. Geoderma.

[bb0170] Hengl T., Kempen B., Heuvelink G.B.M., Malone B. (2015). GSIF: Global Soil Information Facilities. R Package, Version 04-7. https://cran.r-project.org/web/packages/GSIF/.

[bb0175] Hengl T., Heuvelink G.B.M., Kempen B., Leenaars J.G.B., Walsh M.G., Shepherd K., Sila A., MacMillan R.A., Mendes de Jesus J., Tamene L., Tondoh J.E. (2015). Mapping soil properties of Africa at 250 m resolution: random forests significantly improve current predictions. PLoS One.

[bb0180] Hengl T., Mendes de Jesus J.S., Heuvelink G.B.M., Ruiperez Gonzalez M., Leenaars J.G.B. (2017). SoilGrids250m: global gridded soil information based on machine learning. PLoS One.

[bb0185] Hengl T., Leenaars J.G.B., Shepherd K.D., Walsh M.G., Heuvelink G.B.M., Mamo Tekalign, Tilahun Helina, Berkhout E., Cooper M., Fegraus E., Wheeler I., Kwabena Nketia A. (2017). Soil nutrient maps of Sub-Saharan Africa: assessment of soil nutrient content at 250 m spatial resolution using machine learning. Nutr. Cycl. Agroecosyst..

[bb0190] Heuvelink G.B.M. (1998). Error Propagation in Environmental Modelling with GIS.

[bb0195] Heuvelink G.B.M., Arrouays (2014). Uncertainty Quantification of GlobalSoilMap Products.

[bb0200] Heuvelink G.B.M., Pebesma E.J. (1999). Spatial aggregation and soil process modelling. Geoderma.

[bb0205] Hiebert (1974). Risk, learning, and the adoption of fertilizer responsive seed varieties. Am. J. Agric. Econ..

[bb0210] Hodnett M.G., Tomasella J. (2002). Marked differences between van Genuchten soil moisture-retention parameters for temperate and tropical soils: a new water retention pedo-transfer function developed for tropical soils. Geoderma.

[bb0215] ISRIC (2013). Soil Property Maps of Africa at 1 Km. http://www.isric.org/projects/soil-property-maps-africa-1-km-resolution.

[bb0220] IUSS Working Group WRB (2015). International soil classification system for naming soils and creating legends for soil maps. World Soil Resources Reports No. 106. World Reference Base for Soil Resources 2014, Update 2015.

[bb0225] Jayne T.S., Mather D., Mghenyi E. (2010). Principal challenges confronting smallholder agriculture in sub-Saharan Africa. World Dev..

[bb0230] Jeong J.H., Resop J.P., Mueller N.D., Fleisher D.H., Yun K., Butler E.E. (2016). Random forests for global and regional crop yield predictions. PLoS One.

[bb0235] Jones C.A. (1983). Effect of soil texture on critical bulk densities for root growth. Soil Sci. Soc. Am. J..

[bb0240] Jones J.W., Hoogenboom G., Porter C.H. (2003). The DSSAT cropping system model. Eur. J. Agron..

[bb0245] Jones A., Breuning-Madsen H., Brossard M., Dampha A., Deckers J. (2013). Soil Atlas of Africa. European Commission.

[bb0250] Kaufmann M., Tobia S., Schulin R. (2009). Quality evaluation of restored soils with a fuzzy logic expert system. Geoderma.

[bb0255] Kiniry L.N., Scrivner C.L., Keener M.E. (1983). A soil productivity index based upon predicted water depletion and root growth. Research Bulletin 1051.

[bb0260] Lagacherie P., McBratney A.B., Voltz M. (2006). Dev. Soil Sci..

[bb0265] Landon J.R. (1991). Booker tropical soil manual. A Handbook for Soil Survey and Agricultural Land Evaluation in the Tropics and Subtropics.

[bb9005] Leenaars J.G.B. (2012). Africa Soil Profiles Database, Version 1.0. ISRIC Report 2012/03. Africa Soil Information Service (AfSIS) Project.

[bb0270] Leenaars J.G.B., van Oostrum A.J.M., Ruiperez González M. (2014). Africa Soil Profiles Database, Version 1.2. A compilation of georeferenced and standardised legacy soil profile data for Sub-Saharan Africa (with dataset), ISRIC Report 2014/01. Africa Soil Information Service (AfSIS) Project. http://www.isric.org/projects/africa-soil-profiles-database-afsp.

[bb0275] Leenaars J.G.B., B. Kempen, A.J.M. van Oostrum and N.H. Batjes, 2014. Africa Soil Profiles Database: A Compilation of Georeferenced and Standardised Legacy Soil Profile Data for Sub-Saharan Africa. In: Arrouays et al. (eds.), 2014b, 51–57.

[bb0280] Leenaars J.G.B., Hengl T., Ruiperez González M., Mendes de Jesus J.S., Heuvelink G.B.M., Wolf J., van Bussel L.G.J., Claessens L., Yang H., Cassman K.G. (2015). Root Zone Plant-Available Water Holding Capacity of the Sub-Saharan Africa Soil (Dataset RZ-PAWHC SSA v. 1.0). ISRIC Report 2015/02.

[bb0285] Marenya P.P., Barrett C. (2007). Household-level determinants of adoption of improved natural resources management practices among smallholder farmers in western Kenya. Food Policy.

[bb0290] Martens B., Miralles D.G., Lievens H., Schalie R.V.D., de Jeu R.A.M. (2017). GLEAM v3: satellite-based land evaporation and root-zone soil moisture. Geosci. Model Dev..

[bb0295] McBratney A.B., Minasny B., Cattle S.R., Vervoort R.W. (2002). From pedotransfer functions to soil inference systems. Geoderma.

[bb0300] McBratney A.B., Mendonça M.L., Minasny B. (2003). On digital soil mapping. Geoderma.

[bb0305] Minasny B., Malone B.P., McBratney A.B. (2012). Digital Soil Assessments and beyond: Proceedings of the 5th Global Workshop on Digital Soil Mapping 2012.

[bb0310] Morris J.C., Minasny B., McBratney A.B., Minasny (2012). The Role of Soil Inference Systems in Digital Soil Assessments.

[bb0315] Mulders M.A., Leenaars J.G.B., Belemviré A., van Rheenen T., Stroosnijder L. (2001). Soil resources in Sahelian Villages. Agro-Silvo-Pastoral Land Use in Sahelian Villages. Advances in Geo-Ecology.

[bb0320] Pretty J., Toulmin C., Williams S. (2011). Sustainable intensification in African agriculture. Int. J. Agric. Sustain..

[bb0325] Rijsberman F.R., Wolman M.G. (1985). Effects of erosion on soil productivity: an international comparison. J. Soil Water Conserv..

[bb0330] Rötter R., van Keulen H. (1997). Variations in yield response to fertilizer application in the tropics: II. Risks and opportunities for smallholders cultivating maize on Kenya's arable land. Agric. Syst..

[bb0335] Sanchez P.A., Couto W., Buol S.W. (1982). The fertility capability soil classification system: interpretation, applicability and modification. Geoderma.

[bb0340] Sanchez P.A., Palm C.A., Buol S.W. (2003). Fertility capability classification: a tool to help assess soil quality in the tropics. Geoderma.

[bb0345] Shangguan W., Hengl T., de Jesus J.M., Yuan H., Dai Y. (2017). Mapping the global depth to bedrock for land surface modeling. J. Adv. Model. Earth Syst..

[bb0350] Sila A., Hengl T., Terhoeven-Urselmans T. (2014). Package ‘soil.spec’. Soil spectrometry tools and reference models. Africa Soil Information Service.

[bb0355] Soil Survey Division Staff (1993). Soil survey manual. Soil Conservation Service, US Department of Agriculture Handbook 18.

[bb0360] Spitters C.J.T., Schapendonk A.H.C.M. (1990). Evaluation of breeding strategies for drought tolerance in potato by means of crop growth simulation. Plant Soil.

[bb0365] Sys C., van Ranst E., Debaveye J., Beernaert F. (1993). Land evaluation (part III, crop requirements). Agricultural Publications No 7.

[bb0370] Trabucco A., Zomer R.J. (2009). Global Aridity Index (Global-Aridity) and Global Potential Evapo-Transpiration (Global-PET) Geospatial Database. http://www.csi.cgiar.org.

[bb0375] USGS Rocky Mountain Geographic Science Center (2009). African surficial lithology. Africa Land Surface Forms.

[bb0380] Vågen T.G., Shepherd K.D., Walsh M.G., Winowiecki L.A., Desta L.T., Tondoh J.E. (2010). AfSIS technical specifications: soil health surveillance. Africa Soil Information Service (AfSIS).

[bb0385] Vågen T.G., Winowiecki L.A., Tondoh J.E., Desta L.T., Gumbricht T. (2016). Mapping of soil properties and land degradation risk in Africa using MODIS reflectance. Geoderma.

[bb0390] van Diepen C.A., Wolf J., van Keulen H., Rappoldt C. (1989). WOFOST: a simulation model of crop production. Soil Use Manag..

[bb0395] van Genuchten M.T. (1980). A closed-form equation for predicting the hydraulic conductivity of unsaturated soils. Soil Sci. Soc. Am. J..

[bb0400] van Ittersum M.K., Cassman K.G., Grassini P., Wolf J., Tittonell P., Hochman Z. (2013). Yield gap analysis with local to global relevance—a review. Field Crop Res..

[bb0405] van Ittersum M.K., van Bussel L.G.J., Wolf J., Grassini P., van Wart J. (2016). Can sub-Saharan Africa feed itself?. Proc. Natl. Acad. Sci. U. S. A..

[bb0410] van Keulen H., Wolf J. (1986). Modelling of Agricultural Production: Weather, Soils and Crops. Simulation Monographs.

[bb0415] Vanlauwe B., Bationo A., Chianu J., Giller K.E., Merckx R. (2010). Integrated soil fertility management: operational definition and consequences for implementation and dissemination. Outlook Agr..

[bb0420] Vann J., Bertoli O., Jackson S. (2002). An overview of geostatistical simulations for quantifying risk. Proc. Geostat Assoc Australasia ‘Quantifying Risk and Error’.

[bb0425] Webster R., Oliver M.A. (2007). Geostatistics for Environmental Scientists.

[bb0430] Wösten J.H.M., Verzandvoort S.J.E., Leenaars J.G.B., Hoogland T., Wesseling J.G. (2013). Soil hydraulic information for river basin studies in semi-arid regions. Geoderma.

[bb0435] Yang H.S., Dobermann A., Lindquist J.L., Walters D.T., Arkebauer T.J., Cassman K.G. (2004). Hybrid-maize – A Maize Simulation Model that Combines Two Crop Modelling Approaches.

[bb0440] You J., Li X., Low M., Lobell D., Ermon S. (2017). Deep Gaussian Process for Crop Yield Prediction Based on Remote Sensing Data.

[bb0445] Zingore S., Murwira H.K., Delve R.J., Giller K.E. (2007). Soil type, management history and current resource allocation: three dimensions regulating variability in crop productivity on African smallholder farms. Field Crop Res..

